# Correction: Measuring the fitted filtration efficiency of cloth masks, medical masks and respirators

**DOI:** 10.1371/journal.pone.0330227

**Published:** 2025-08-12

**Authors:** Amanda A. Tomkins, Gurleen Dulai, Ranmeet Dulai, Sarah Rassenberg, Darren Lawless, Scott Laengert, Rebecca S. Rudman, Shiblul Hasan, Charles-Francois de Lannoy, Ken G. Drouillard, Catherine M. Clase

There are errors in the Author Contributions. The correct contributions are:

**Conceptualization:** Amanda A Tomkins, Gurleen Dulai, Ranmeet Dulai, Sarah Rassenberg, Darren Lawless, Scott Laengert, Rebecca S Rudman, Charles-Francois de Lannoy, Ken G Drouillard, Catherine M Clase

**Data curation:** Amanda A Tomkins, Gurleen Dulai, Ranmeet Dulai, Ken G Drouillard, Catherine M Clase

**Formal analysis:** Amanda A Tomkins, Gurleen Dulai, Ranmeet Dulai, Ken G Drouillard, Catherine M Clase

**Investigation:** Amanda A Tomkins, Gurleen Dulai, Ranmeet Dulai, Sarah Rassenberg, Scott Laengert, Rebecca S Rudman, Charles-Francois de Lannoy, Ken G Drouillard, Catherine M Clase

**Methodology:** Amanda A Tomkins, Gurleen Dulai, Ranmeet Dulai, Sarah Rassenberg, Scott Laengert, Rebecca S Rudman, Charles-Francois de Lannoy, Ken G Drouillard, Catherine M Clase

**Project administration:** Darren Lawless, Rebecca S Rudman, Charles-Francois de Lannoy, Ken G Drouillard, Catherine M Clase

**Resources:** Gurleen Dulai, Ranmeet Dulai, Darren Lawless, Rebecca S Rudman, CharlesFrancois de Lannoy, Ken G Drouillard, Catherine M Clase

**Software:** Ken G Drouillard, Catherine M Clase

**Supervision:** Charles-Francois de Lannoy, Ken G Drouillard, Catherine M Clase

**Validation:** Amanda A Tomkins, Gurleen Dulai, Ranmeet Dulai, Ken G Drouillard, Catherine M Clase

**Visualization:** Amanda A Tomkins, Ranmeet Dulai, Rebecca S Rudman, Shiblul Hasan, Ken G Drouillard, Catherine M Clase

**Writing – original draft:** Amanda A Tomkins, Gurleen Dulai, Ranmeet Dulai, Ken G Drouillard, Catherine M Clase

**Writing – review & editing:** Amanda A Tomkins, Gurleen Dulai, Ranmeet Dulai, Sarah Rassenberg, Darren Lawless, Scott Laengert, Rebecca S Rudman, Shiblul Hasan, CharlesFrancois de Lannoy, Ken G Drouillard, Catherine M Clase
