# Supplementary material for: Measuring the fitted filtration efficiency of cloth masks, medical masks and respirators
Source: PLoS One. 2025 Apr 21;20(4):e0301310. doi: 10.1371/journal.pone.0301310 (PMC12011288; doi:10.1371/journal.pone.0301310)
Supplement: S1 Table — * Face width narrower than smallest face width on the NIOSH panel, classified according to menton-sellion. Facial hair (mask hacks study only) was classified according to CDC NIOSH (24). (PDF) [file pone.0301310.s001.pdf]

S1 Table. Characteristics of the 12 participants, including ethnic origin, facial hair type and NIOSH panel size.

Participant	Types	Hacks	Over-masking	Age Range	Gen der	Facial hair	Facial hair type[19]	Self-identified Ethnic Origin	Height, m	Weight, kg	Bizygomatic distance, calipers, cm	Menton-sellion distance, calipers, cm	Bizygomatic distance, cord, cm	Menton-sellion distance, cord, cm	NIOSH Bivariate Panel Size
1	x	x	x	50-59	F	N	NA	European	1.68	62	13.0	10.0	17.2	13.1	1
2		x		50-59	M	Y	Full beard	European	1.66	70	15.0	9.0	19.0	12.9	5
3		x		20-29	F	N	NA	European	1.78	53	11.6	10.7	15.5	18.6	1
4	x	x	x	20-29	F	N	NA	European	1.77	80	13.0	11.0	15.4	18.2	3
5		x		20-29	M	Y	Stubble	European	1.79		14.9	12.2	15.7	15.2	8
6		x		20-29	F	N	NA	European	1.81	68	14.4	11.9	15.0	17.5	7
7		x		20-29	M	Y	Full Beard	West Central Asian and Middle Eastern	1.83	86	14.6	13.3	16.8	17.2	10
8		x		20-29	M	N	NA	European	1.88	85	13.0	12.0	13.4	14.0	6
9		x	x	50-59	F	N	NA	European	1.65	95	13.3	11.4	11.3	13.3	4
10		x	x	50-59	M	N	NA	European	1.77	91	14.3	13.0	12.2	15.3	9
11	x		x	20-29	F	N	NA	West Central Asian and Middle Eastern	1.61	57	11.5	11.0	15.0	13.0	6*
12	x		x	20-29	F	N	NA	West Central Asian and Middle Eastern	1.6	54	11.5	12.0	15.0	13.6	6*

* Face width narrower than smallest face width on the NIOSH panel, classified according to menton-sellion. Facial hair (mask hacks study only) was classified according to CDC NIOSH (1)
