# Supplementary material for: Measuring the fitted filtration efficiency of cloth masks, medical masks and respirators
Source: PLoS One. 2025 Apr 21;20(4):e0301310. doi: 10.1371/journal.pone.0301310 (PMC12011288; doi:10.1371/journal.pone.0301310)
Supplement: S2 Table — Leaks, Glasses Fog and Comfort are also shown graphically in S3-5 Figs. * 1 Moving into eyes, 2 Moving around, not into eyes, 3 Straps pinching, 4 Nose stuffy, 5 Hard to breathe, 6 Interferes with hair/head covering; multiple occurrences of the same number indicate that multiple participants reported the same issue. FTM Fix the Mask. (PDF) [file pone.0301310.s002.pdf]

S2 Table. Subjective data for the mask hacks substudy; top panel, level 1 ASTM certified mask; bottom panel, level 3 ASTM certified mask. Leaks, Glasses Fog and Comfort are also shown graphically in S3-5 Figs.

L1					
Mask	Leaks	Glasses Fog	Comfort	Issues*	Comments
L1 Alone	6, 5, 4, 2, 2, 4, 7, 7	7, 6, 4, 2, 5, 6, 7, 6	2, 1, 2, 2, 2, 3, 5, 2	1, 1, 2, 2, 2, 2, 1	"riding on face, elastic broke", "material is itchy, mask moved around a lot"
Ear Saver	4, 3, 4, 2, 2, 3, 4, 3	6, 6, 4, 2, 6, 6, 7, 7	2, 1, 2, 3, 3, 3, 5, 3	1, 2, 2, 6, 6, 6	"guard slides a lot on head/hair", "itchy, stayed in place, easy to put on"
Scrub Cap	5, 5, 3, 3, 3, 4, 5, 5	6, 7, 4, 1, 6, 4, 6, 7	2, 2, 3, 1, 2, 2, 5, 2	1, 2, 1	"major leak at nose"
Knot and Tuck	3, 3, 2, 1, 4, 2, 3, 1	3, 7, 6, 1, 2, 3, 4, 1	3, 4, 3, 2, 6, 3, 4, 3	1, 2, 1, 2	"did not feel secure, had to adjust", "smaller, harder to fit in", "had to reposition during bending", "does not fit face well", "does not fully fit", "fell down twice during talking"
Silicone Brace	2, 1, 1, 1, 3, 3, 1, 1	2, 2, 2, 2, 4, 6, 1, 1	4, 2, 3, 3, 4, 4, 3, 4	3, 6, 4	"wire could be helpful", "pulled on nose", "tight on nose", "hard to put on, comfortable", "straps hard to adjust"
Simple Brace	3, 3, 3, 3, 3, 3, 1, 3	3, 6, 2, 2, 4, 6, 1, 1	5, 6, 4, 5, 7, 5, 7, 5	2, 3, 4, 2, 4, 5, 6, 4	"nose fit poor, kept slipping", "pressure on nose", "pushed up on nose", "leak on nose", "heavy on face, choking, tight on chin", "minor/slight leak at nose"
FTM Brace	2, 2, 1, 1, 1, 2, 4, 1	2, 2, 1, 1, 3, 2, 4, 1	5, 6, 4, 3, 7, 4, 6, 4	3, 4, 6, 4, 6, 3, 2, 5, 6, 2, 5	"pressure on sinuses/area around nose", "nose fingers moving, bottom slides around", "hard to breathe through nose", "hard to put on, choking, pinches eyes, 'octopus of death'", "nubs are hard to smooth out"
Essex Pleated	4, 5, 5, 2, 2, 3, 3, 3	7, 7, 6, 4, 2, 6, 2, 2	4, 4, 3, 2, 6, 2, 4, 3	1, 3, 4, 2, 2	"loops folded ears", "slid on nose", "inner mask slipped"

*1 Moving into eyes, 2 Moving around, not into eyes, 3 Straps pinching, 4 Nose stuffy, 5 Hard to breathe, 6 Interferes with hair/head covering; multiple occurrences of the same number indicate that multiple participants reported the same issue. FTM Fix the Mask.

L3					
Mask	GF	Leaks	Comfort	Issues	Comments
L3 Alone	3, 3, 1, 2, 3, 3, 4, 1	3, 7, 1, 2, 4, 2, 4, 1	2, 2, 1, 2, 1, 2, 3, 2	1, 1, 2, 2	“leak on cheek”, “really good fit on nose”, “itchy”
Ear Saver	2, 3, 1, 3, 1, 2, 3, 1	3, 7, 2, 2, 4, 2, 1, 1	2, 1, 1, 3, 2, 3, 4, 1	1, 2, 6, 6	“folded up”, “itchy on nose”, “itchy”
Scrub Cap	4, 2, 1, 2, 2, 3, 3, 1	4, 4, 6, 2, 2, 2, 4, 1	3, 1, 3, 3, 1, 3, 3, 2	2, 2	“moved on chin”, “pulling away from the face”
Knot and Tuck	2, 3, 3, 1, 2, 4, 3, 1	2, 2, 4, 2, 1, 1, 2, 1	2, 1, 5, 3, 2, 3, 5, 3	2, 2, 2	“mask too small, moved when talking”, “too small so pockets were created on sides”, “hot, itchy”
Silicone Brace	1, 1, 1, 2, 1, 2, 1, 1	2, 1, 2, 1, 2, 2, 1, 1	3, 2, 3, 3, 3, 5, 3, 3	6	“itchy, stays in place”
Simple Brace	3, 2, 1, 2, 3, 2, 1, 1	3, 1, 1, 2, 2, 4, 1, 1	5, 6, 3, 3, 7, 5, 7, 5	4, 2, 5, 4, 6, 3, 4	“leak at nose, tight”, “hard to get on”, “very tight” “tight on nose”, “hot, itchy, chokes”
FTM Brace	2, 2, 1, 1, 2, 2, 3, 1	2, 2, 2, 1, 3, 2, 2, 1	4, 7, 3, 4, 5, 5, 6, 3	3, 4, 5, 6, 4, 6, 2, 4, 4	“pleat folded”, “straps undid but still secure, pushing on nose”, “itchy, moves around, hot”, “nose pieces moved”
Essex Pleated	2, 5, 2, 2, 1, 4, 3, 1	3, 7, 6, 2, 1, 1, 2, 2	4, 2, 3, 3, 6, 3, 3, 1	1, 1, 3, 2	“elastic hurts, Essex slides in eyes”

*1 Moving into eyes, 2 Moving around, not into eyes, 3 Straps pinching, 4 Nose stuffy, 5 Hard to breathe, 6 Interferes with hair/head covering; multiple occurrences of the same number indicate that multiple participants reported the same issue. FTM Fix the Mask
