# Supplementary material for: Measuring the fitted filtration efficiency of cloth masks, medical masks and respirators
Source: PLoS One. 2025 Apr 21;20(4):e0301310. doi: 10.1371/journal.pone.0301310 (PMC12011288; doi:10.1371/journal.pone.0301310)
Supplement: S3 Table — Leaks, Glasses Fog and Comfort are shown graphically in S3-5 Figs. * 1 Moving into eyes, 2 Moving around, not into eyes, 3 Straps pinching, 4 Nose stuffy, 5 Hard to breathe, 6 Interferes with hair/head covering Multiple occurrences of the same number indicate that multiple participants reported the same issue. L1 level 1; L3 level 3; EL Earloops; FT Fabric ties. (PDF) [file pone.0301310.s003.pdf]

S3 Table. Subjective data for the overmasking study; top panel, level 1 and level 3 ASTM certified mask. Leaks, Glasses Fog and Comfort are shown graphically in S3-5 Figs.

Mask	Leaks	Glasses Fog	Comfort	Issues	Comments
L1	6, 3.3, 4	5, 1, 2	2.5, 1, 2	1, 1	
L1 + Essex Pleated (EL)	2.7, 1.7, 2	5.5, 1.3, 1.7	2.5, 6, 3	1, 1, 3, 4, 5, 1, 5	"leaks only at nose"
L1 + Essex Pleated (FT)	2.7, 1, 1.3	4.7, 1, 1.8	3, 3, 6	6, 1, 6, 3, 4, 5, 6	"inner mask touches lips with and without talking, leaks only at nose, feels snug, material contacts mouth at rest and it's noticeable with talking"
L3	2, 3, 3	3, 1.7, 1.8	2, 1.3, 2	1, 1	
L3 + Essex Pleated (EL)	1.7, 2, 2	2.6, 1.2, 1.7	3.5, 6, 3.7	1, 3, 4, 5, 1, 5	"more comfortable than a brace, quite warm, leaks only at nose"
L3 + Essex Pleated (FT)	2, 1, 1	3.8, 1, 1.5	3, 3, 6.7	6, 1, 6, 1, 4, 5, 6	"inner material contacts mouth at rest and when talking, fabric touches face but in general comfortable, doesn't pinch or pull anywhere, very stable"
Essex Pleated (EL)	3.7, 3.3, 2.3	5.25, 1.3, 1.8	2.3, 4.7, 3	1, 1, 3, 4, 1, 2, 3	"feels high on face, in eyes a bit, stable, leaks only at nose, pulls a little on ears, pulls down tip of nose"
Essex Pleated (FT)	3.3, 2, 1.3	5.3, 1, 1.2	2, 3, 6.7	6, 1, 6, 1, 4, 5, 6	"stays put, very secure and doesn't move, material doesn't contact lips"
Essex Pleated (EL) + Essex Pleated (EL)	2.7, 2.3, 3	5, 1, 1.3	2.7, 6, 4	1, 3, 4, 2, 3, 5	"the double elastic with the wide elastic doesn't feel very secure on ears, hot, stable, comfortable, leaks only at nose, pulls a little on ears, pulls down tip of nose, doesn't move but outer mask sits in eyes"
Essex Pleated (EL) + Essex Pleated (FT)	2.3, 2, 1	4.1, 1, 1.3	2.7, 3, 6.3	6, 1, 6, 3, 4, 5, 6	"no movement at all, tugs down on tip of nose, barely any leak even at eyes, warm, fabric contacts mouth at rest and more noticeable with speaking, pulls down on the point of nose with head up position and neck extension, warm but not excessively for sedentary activities like these"

*1 Moving into eyes, 2 Moving around, not into eyes, 3 Straps pinching, 4 Nose stuffy, 5 Hard to breathe, 6 Interferes with hair/head covering

Multiple occurrences of the same number indicate that multiple participants reported the same issue.

L1 level 1; L3 level 3; EL Earloops; FT Fabric ties
