# Supplementary material for: Measuring the fitted filtration efficiency of cloth masks, medical masks and respirators
Source: PLoS One. 2025 Apr 21;20(4):e0301310. doi: 10.1371/journal.pone.0301310 (PMC12011288; doi:10.1371/journal.pone.0301310)
Supplement: S1 Fig — (PDF) [file pone.0301310.s004.pdf]

S1 Fig. Descriptions and photographs of representative masks and of minor modifications (hacks) to improve fitted filtration efficiency of masks.

**Essex Pleated on Earloops**	Two-layer 3-pleated mask made using 100% woven cotton. The pattern was designed by a consensus panel for the Windsor-Essex Sewing Force and has **no nose-wire**, and side channels for head attachments - in this case **¼ inch elastic earloops**.<br>Robert Koffman brand "Science Fair" fabric; 100% cotton with plain weave; 135 threads per inch; 157.9±4.3 g/m2 fabric weight; mean pore diameter 91.8±23.2 µm; 4.63±0.13 pressure differential (mm of H2O/cm2). Detailed pattern and instructions available as 'Essex mask' at https://maskevidence.org/patternsinstruction		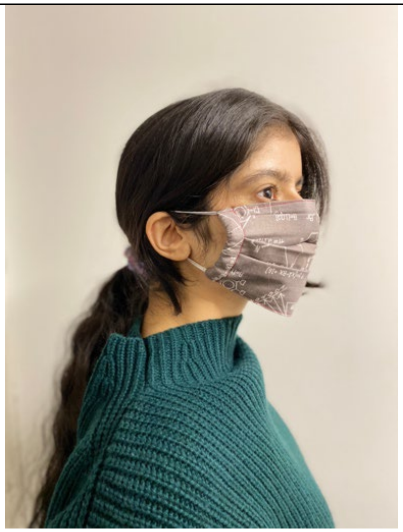
**Essex Pleated on Overhead Ties**	Two-layer 3-pleated mask made using 100% woven cotton. The pattern was designed by a consensus panel for the Windsor-Essex Sewing Force and has **no nose-wire**, and side channels for head attachments - in this case **cloth overhead ties**. Robert Koffman brand "Science Fair" fabric; 100% cotton with plain weave; 135 threads per inch; 157.9±4.3 g/m2 fabric weight; mean pore diameter 91.8±23.2 µm; 4.63±0.13 pressure differential (mm of H2O/cm2). Detailed pattern and instructions available as 'Essex mask' at https://maskevidence.org/patternsinstruction	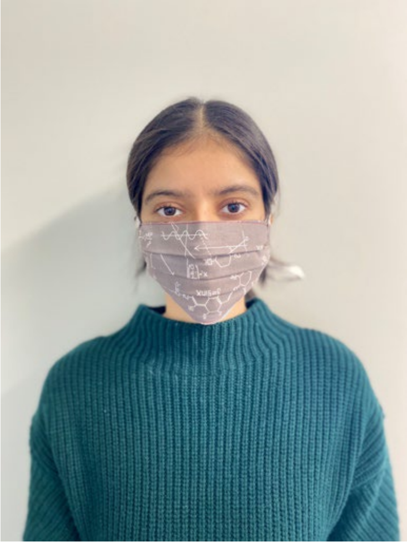	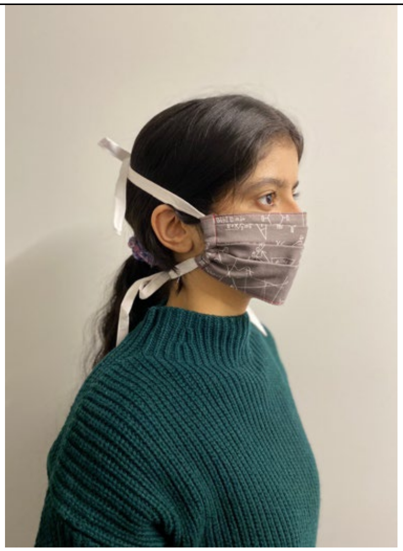

**Level 1 and 3 Certified Masks**	Masks certified according to the standards of ASTM International: level 1 masks (Polar Bear and O2) and level 3 masks (Halyard and Primed). All the certified masks we used were earloop masks. Shown: Level 1 Polar Bear. All four masks have nosewires.	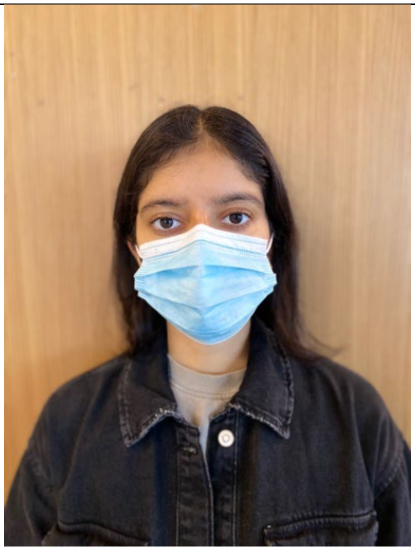	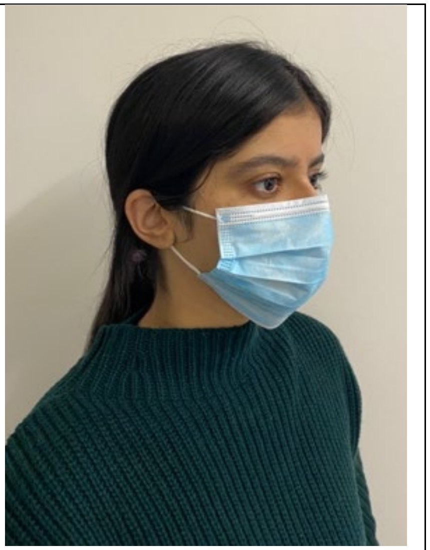
**KF94, KN95, and Similar**	These were sourced from a purposive online search 2022 Jan 03. Left panel shows representative 3D design (KF94 BuyEverything, KF94 Fimaly, Kegis 3D) and right panel shows representative bifold design (KN95 Chengde, KN95 GZHarly, KN95 Goltum). Shown, left: KF94 BuyEverything; right: KN95 Chengde. **All masks have nosewires.**	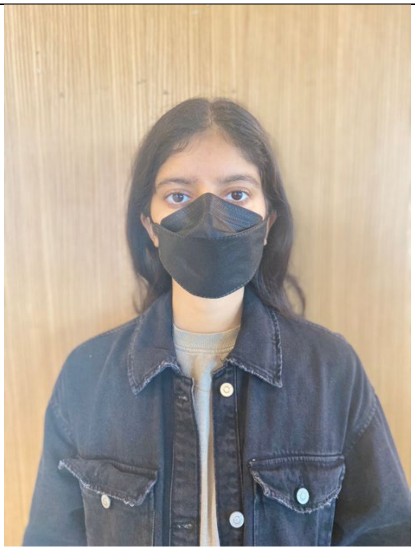	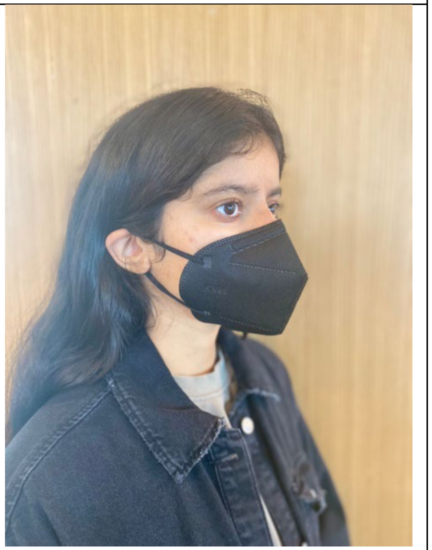

**Respirators**	NIOSH-certified respirators have overhead attachments. Shown, left: N95 3M Aura 1870; right: CaN99 Vitacore. **All masks have nosewires.** The 3M Aura 1870 also has a **foam pad** beneath the nosewire, which contacts the bridge of the nose.	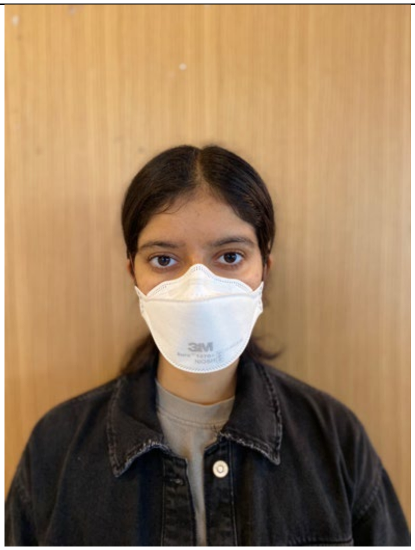	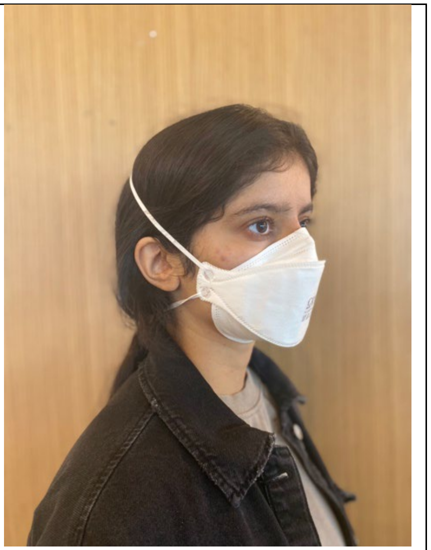
**Earguard**	Earguard (also called ear saver) at the nape of the neck; the earloop passes over the ear and hooks on the earguard, changing the angle of the bottom of the earloop and increasing the tension. The wearer chooses acceptable tension without discomfort.(1)	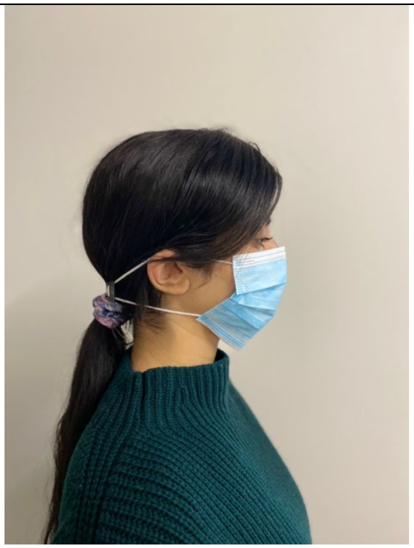	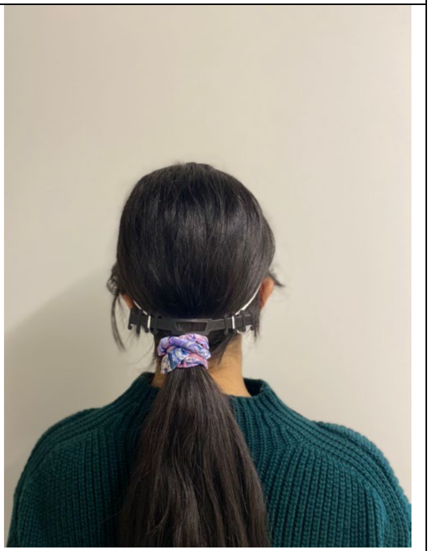

**Scrub Cap**	Scrub cap with buttons. We created scrub caps with multiple buttons at 1 cm intervals. We asked participants to pass the earloop around at least 2 buttons, choosing the placement that gave them the subjective sense of the optimal comfort and fit.	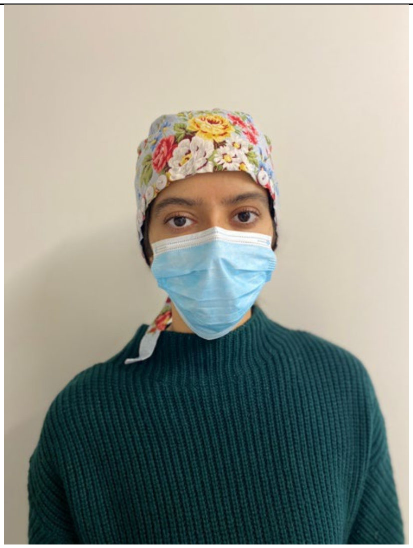	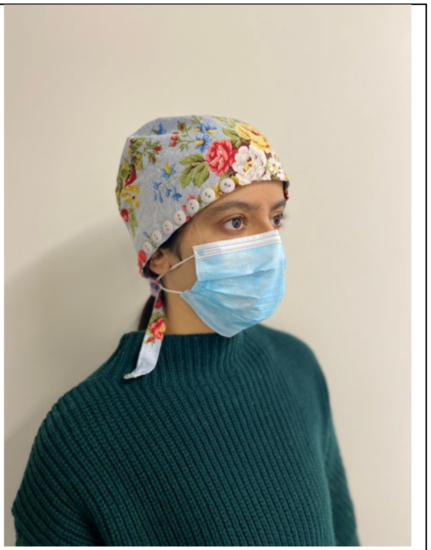
**Knot-and-Tuck**	Knot-and-tuck described by Center for Disease Control(1, 2). The earloop is knotted close to the mask and the excess material folded flat against the face, as described in this video(3)	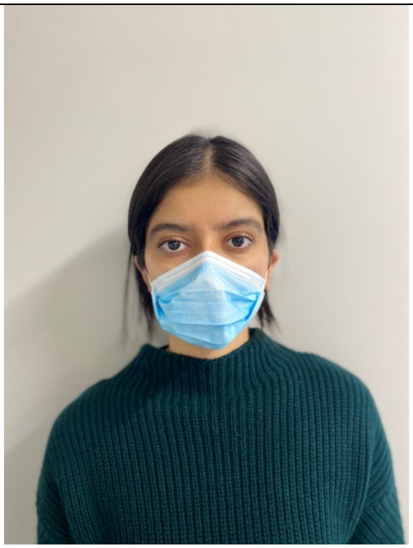	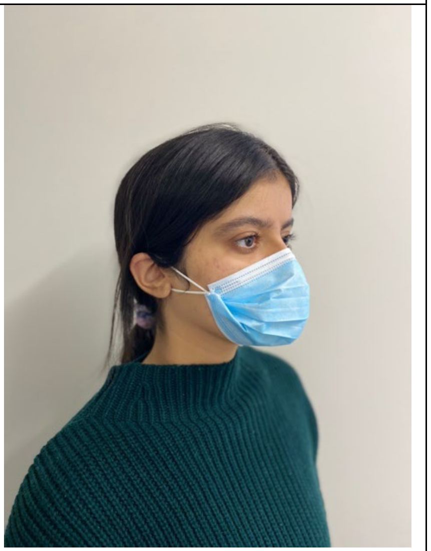

**Neoprene Brace**	Neoprene public-domain brace (N-brace) designed by Fix-the-mask investigators and made from the recommended 1/32" Shore 40A rubber sheets.(4)	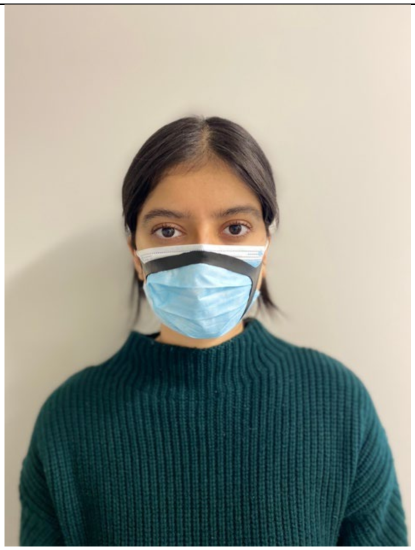	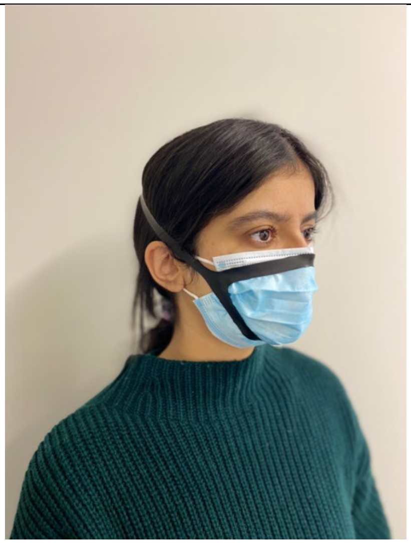
**Silicone Brace**	Silicone brace (S-brace) with broad elastic head attachments designed by Fang, Tomkins et al at CEPEM. The wearer adjusted the straps to obtain the best subjective fit.	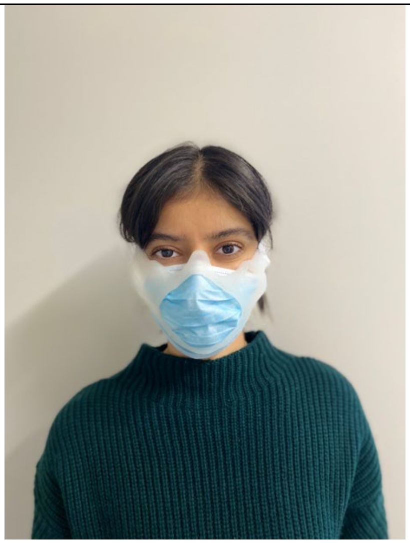	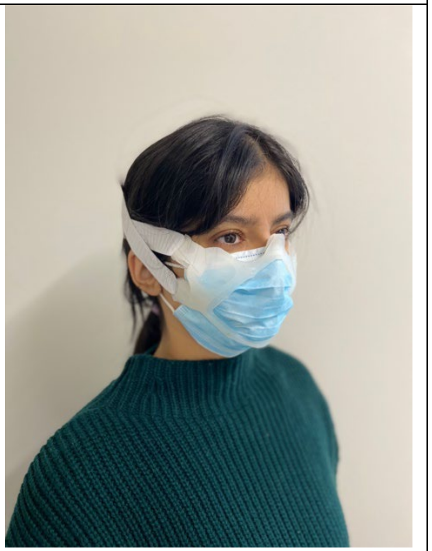

<table>
<tr><td>Fix</td><td>Fix-the-mask brace (FTM brace). These braces have patented nose cushions designed to close the gap under the eyes.(5)</td><td>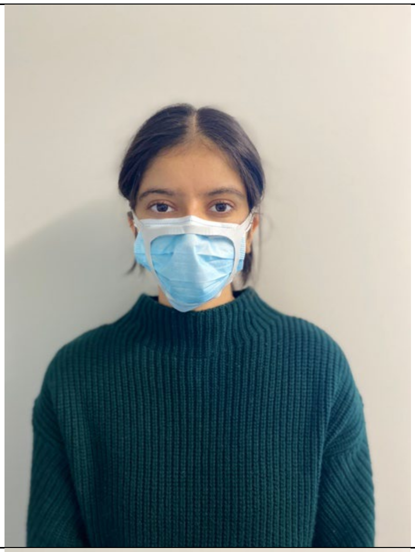</td><td>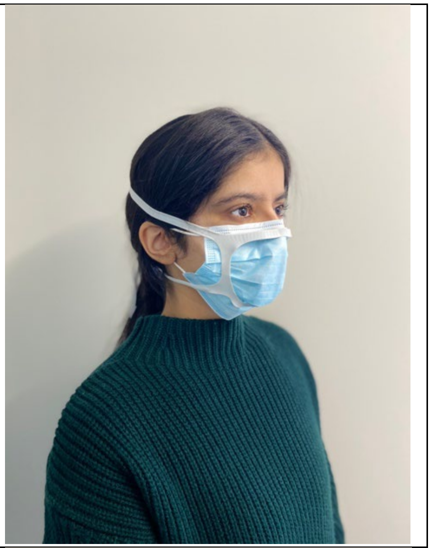</td></tr>
<tr><td>Overmasking</td><td>The Essex pleated (here shown on ties) is worn over the level 1 or level 3 mask (video demonstration at https://wesf.ca/research)</td><td>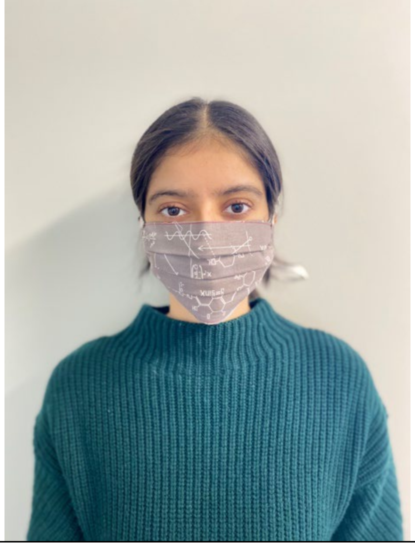</td><td>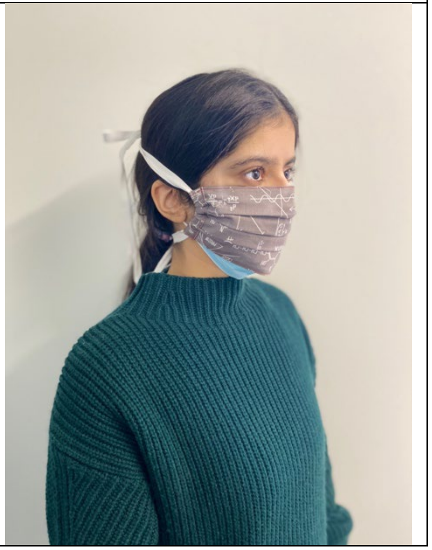</td></tr>
</table>
