# Supplementary figures and images for: Measuring the fitted filtration efficiency of cloth masks, medical masks and respirators
Source: PLoS One. 2025 Apr 21;20(4):e0301310. doi: 10.1371/journal.pone.0301310 (PMC12011288; doi:10.1371/journal.pone.0301310)

S2 Fig. A research participant wearing a cloth mask on ties for testing

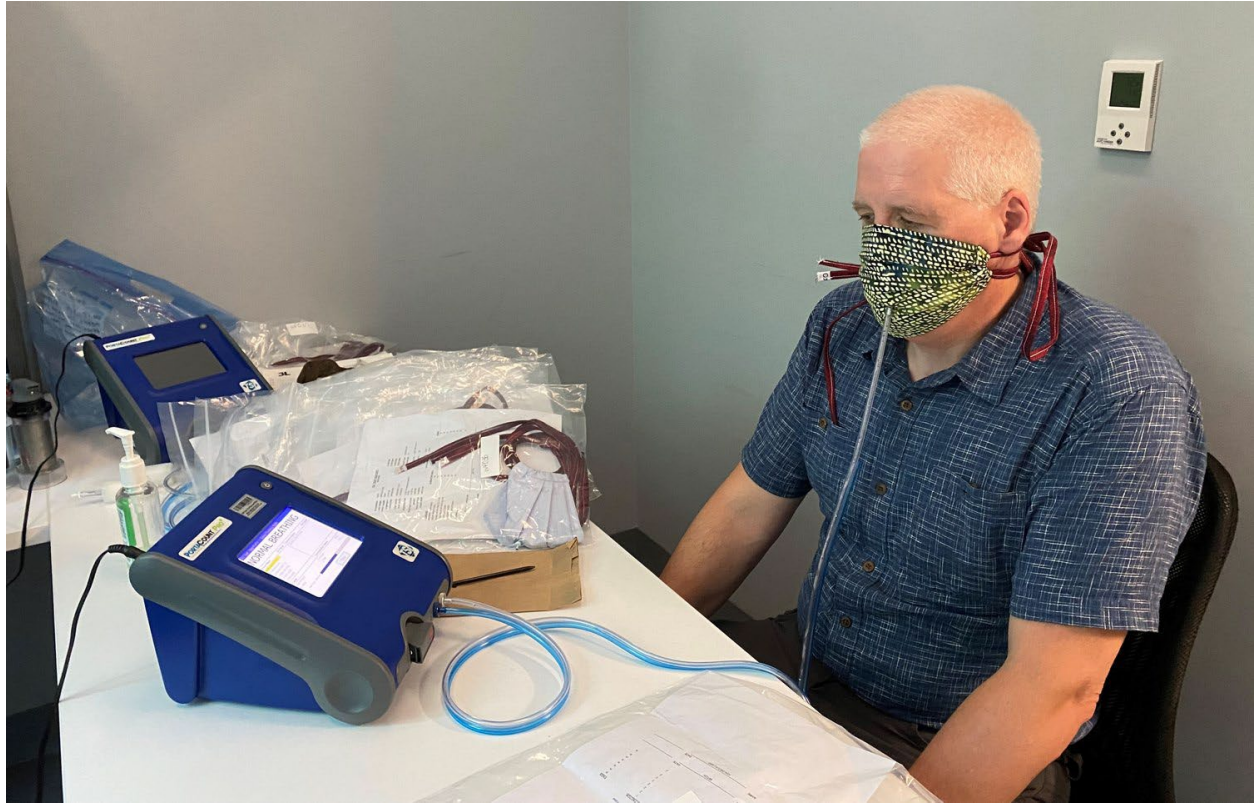

Supplement: S2 Fig — (PDF) [file pone.0301310.s005.pdf]

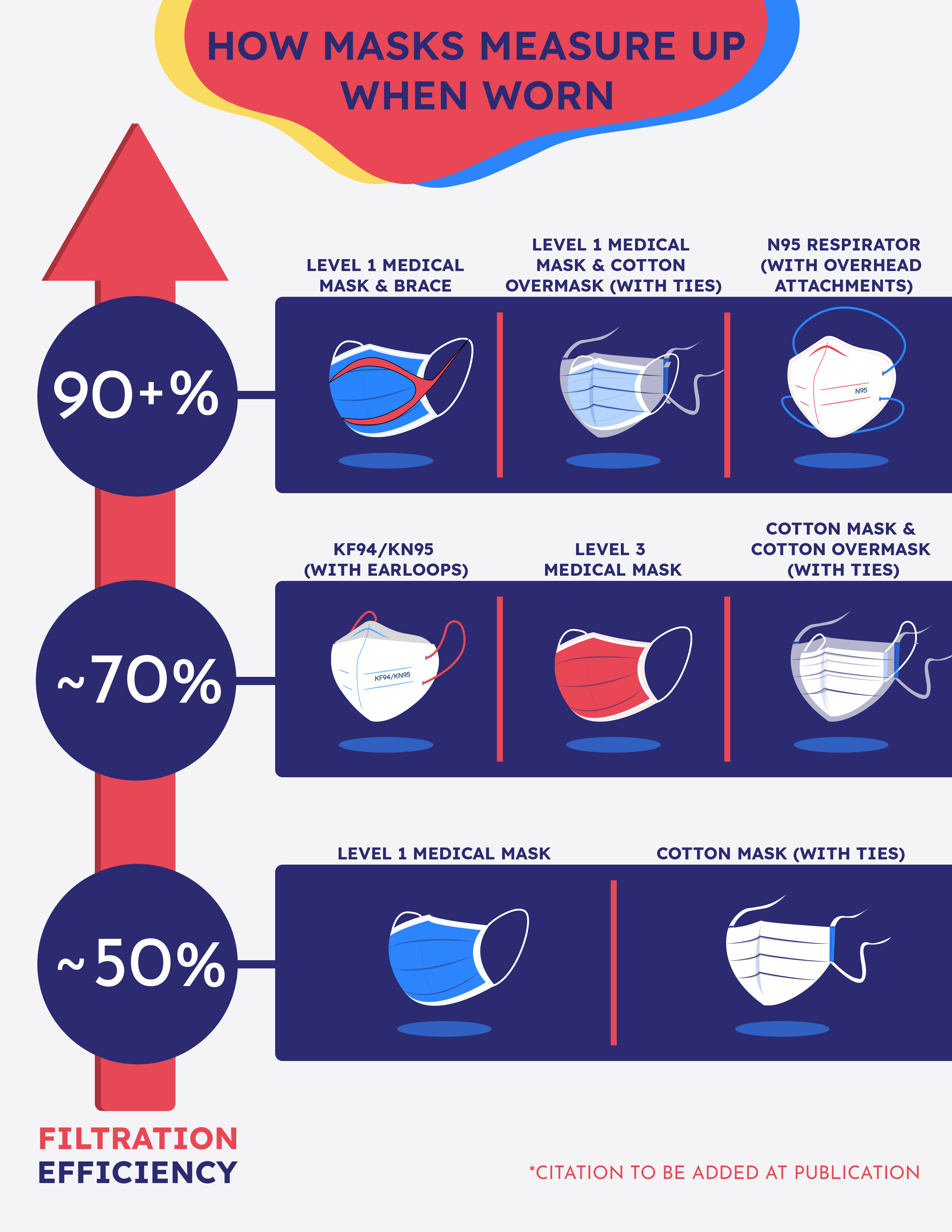

Supplement: S7 Fig — (JPG) [file pone.0301310.s010.jpg]
