# Supplementary material for: Measuring the fitted filtration efficiency of cloth masks, medical masks and respirators
Source: PLoS One. 2025 Apr 21;20(4):e0301310. doi: 10.1371/journal.pone.0301310 (PMC12011288; doi:10.1371/journal.pone.0301310)

S5 Fig. Subjective discomfort scores. Normal when mean shown, otherwise non-normal. Top panel mask types; middle panel mask hacks; bottom panel overmasking.

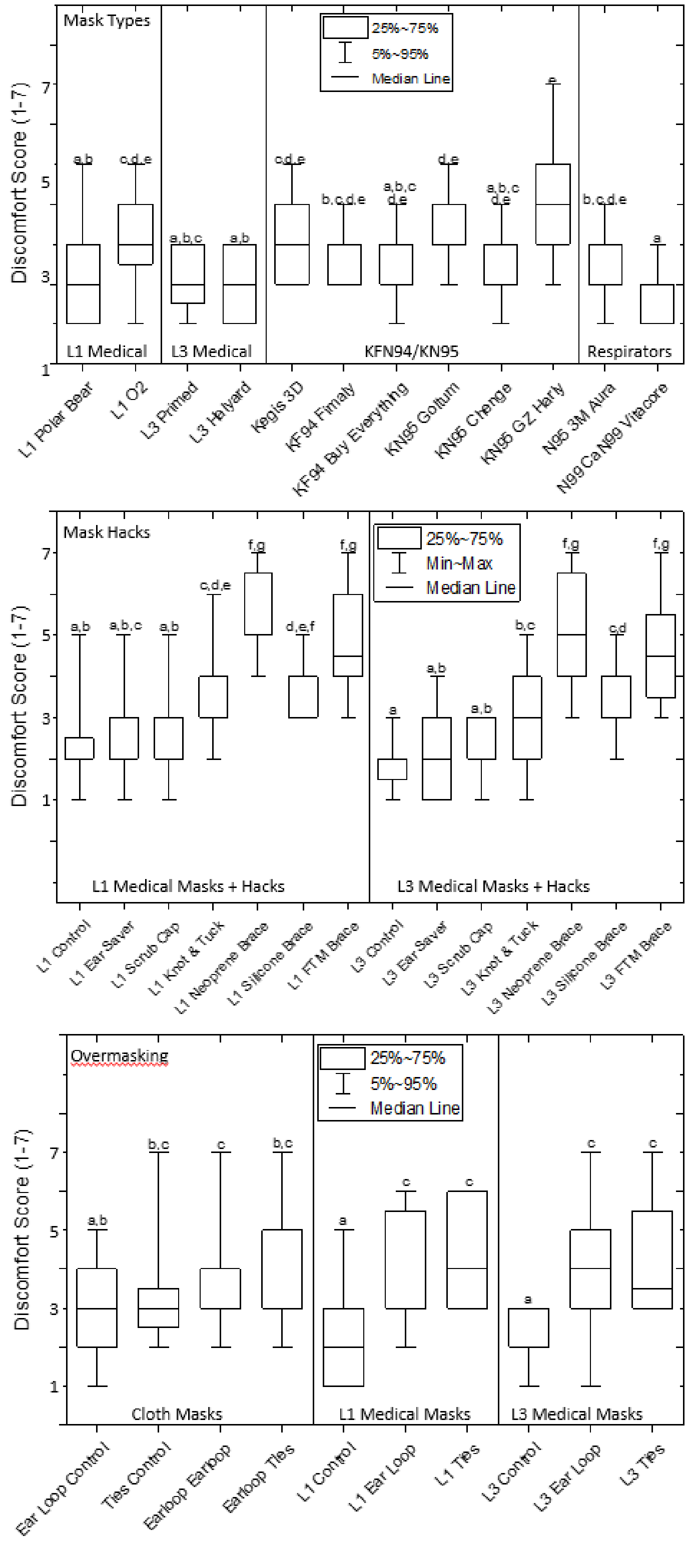

Supplement: S5 Fig — Normal when mean shown, otherwise non-normal. Top panel mask types; middle panel mask hacks; bottom panel overmasking. (PDF) [file pone.0301310.s008.pdf]
