# Supplementary material for: Measuring the fitted filtration efficiency of cloth masks, medical masks and respirators
Source: PLoS One. 2025 Apr 21;20(4):e0301310. doi: 10.1371/journal.pone.0301310 (PMC12011288; doi:10.1371/journal.pone.0301310)
Supplement: S6 Fig — (PDF) [file pone.0301310.s009.pdf]

S6 Fig. Relationship between cord measures and caliper measures, for bizygomatic and for menton-sellion distances, R^2 and p for linear regression; line of best fit not shown because $P > 0.05$ and R^2 small.

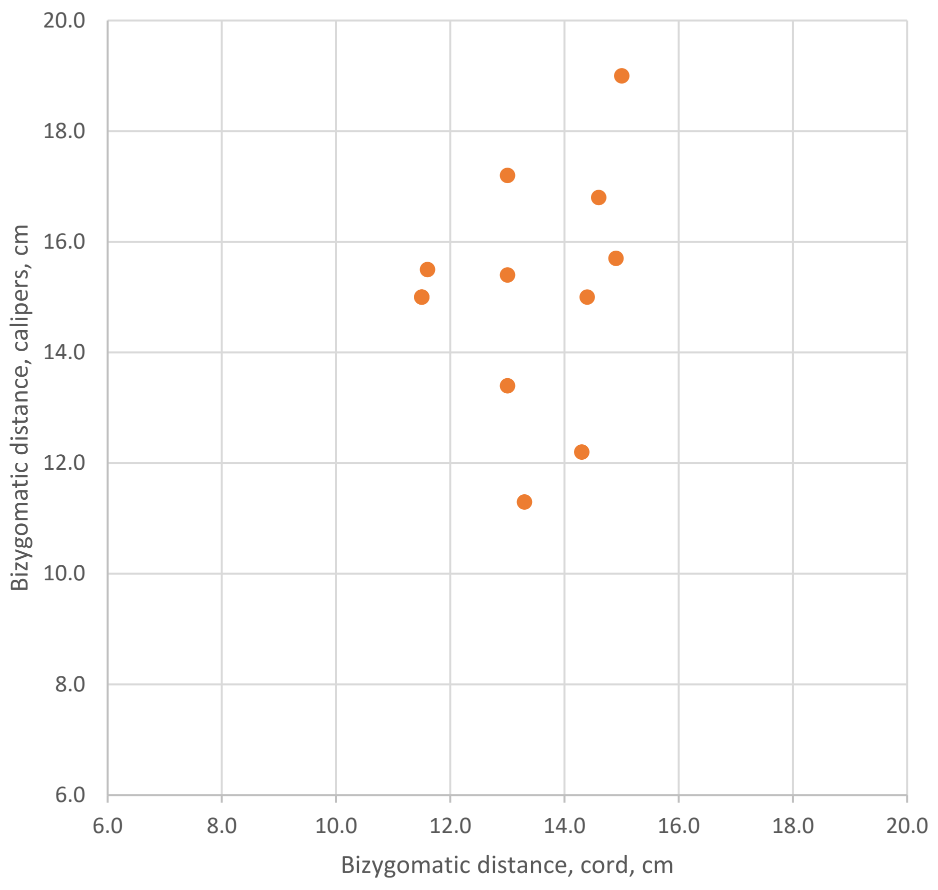

R^2 0.03; $p = 0.53$

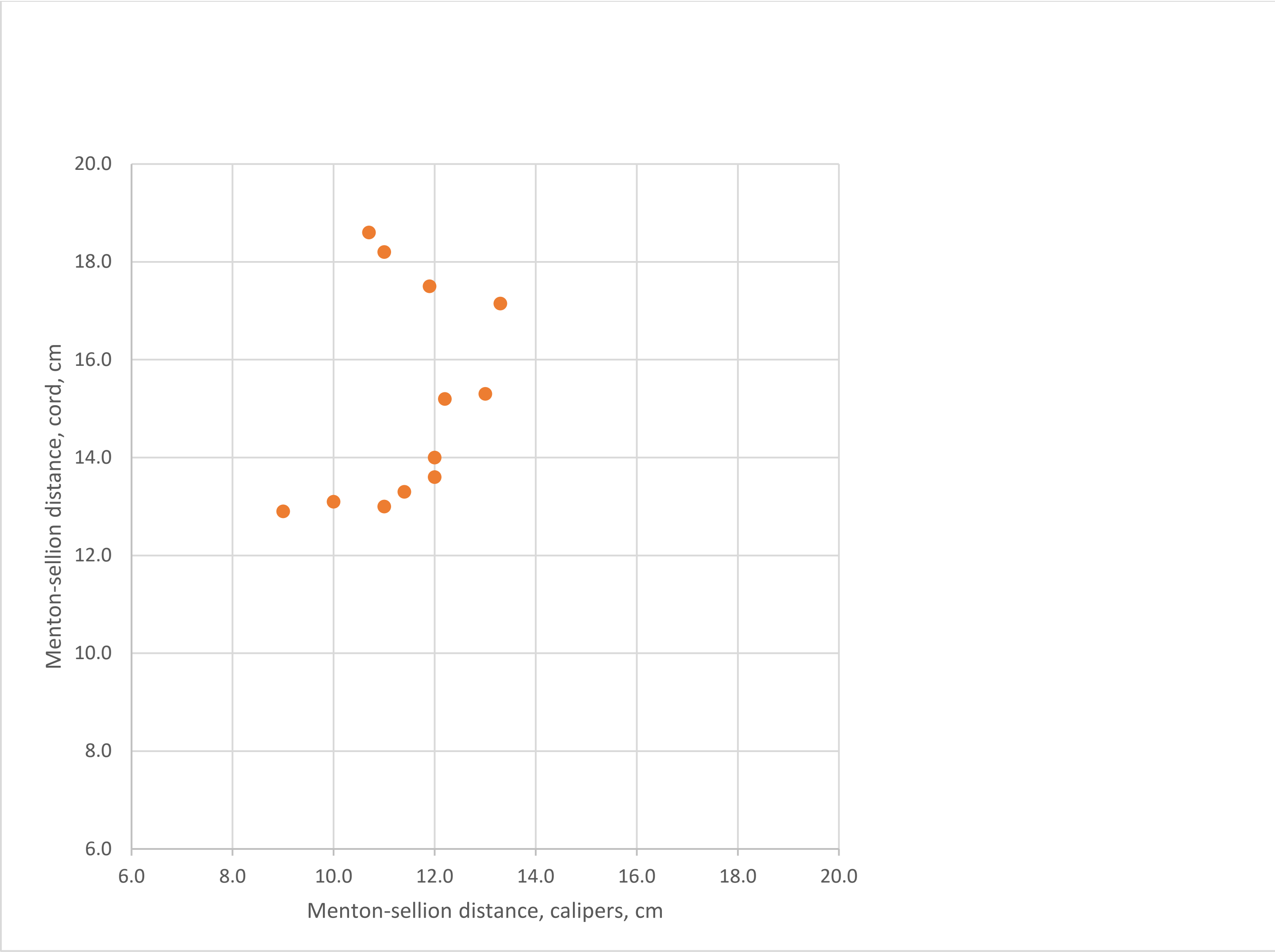

20.0
18.0
16.0
14.0
12.0
10.0
8.0
6.0
Menton-sellion distance, cord, cm
6.0
8.0
10.0
12.0
14.0
16.0
18.0
20.0
Menton-sellion distance, calipers, cm

R^2 0.09; $p = 0.27$
