# Supplementary material for: Measuring the fitted filtration efficiency of cloth masks, medical masks and respirators
Source: PLoS One. 2025 Apr 21;20(4):e0301310. doi: 10.1371/journal.pone.0301310 (PMC12011288; doi:10.1371/journal.pone.0301310)
Supplement: S1 Appendix — (PDF) [file pone.0301310.s011.pdf]

S1 Appendix

Facial Measurement: General

After obtaining informed consent by teleconference, we conducted facial measurements on all participants. We used the bizygomatic breadth and menton-sellion length of each participant to determine their face size according to the specifications of the NIOSH Bivariate Panel of the F3407-20 standard issued by ASTM International, previously known as American Society for Testing and Materials (Supplementary Material: F3407-20 – NIOSH Panel Based on Face Length and Width). ASTM 3502 suggests that NIOSH face sizes of 2-7 and 9 be considered ‘medium’; 1 is ‘small’ and 8, 10 are ‘large’.

We performed measurements according to the Measurer’s Handbook: US Army Anthropometric Survey, 1987-88 as reproduced in Appendix A of the 2004 report of respirator users (1) adhering to a Standard Operating Procedure (SOP, below p9) to mitigate risk of transmission of COVID-19. Briefly, we obtained facial measurements using either hinged calipers or a flexible piece of cord. In the approach using calipers, a researcher used the calipers to capture the dimensions of the participant’s bizygomatic breadth and menton-sellion length. We carefully aligned the spreading calipers with a ruler placed on a flat surface to read the caliper distance. Recognizing that these formal caliper measurements are not available to the general public or to frontline workers, or necessarily the most sensible measurements when considering fit, we also measured the same distances using cord. Following instruction, in person, by zoom, or by video (2) participants aligned the cord across their face, measuring the most direct distance between the landmarks. For bizygomatic measurements, the cord crossed the nose horizontally wherever it naturally fell using comfortable tension (rather than diverting up to the sellion). For menton-sellion measurements, the cord travelled over the tip of the nose. The measurer then carefully aligned the string with a ruler placed on a flat surface.

Minimal facial measurements performed during a pandemic or epidemic

This standard operating procedure (SOP) outlines the methods for obtaining facial measurements in a situation in which either the tester or the participant may an asymptomatic or pre-symptomatic carrier of the pathogen.

The risk of transmission of SARS-CoV-2, the virus that causes COIVD-19 is dependent on proximity, duration, contact of mucous membranes, and the wearing of masks. The same is likely to be true of future pathogens. This SOP describes how to obtain essential data to inform about the fit of masks in the context of this risk, and seeks to minimize the risk.

Of the 20 measurements described in the 2004 head-and-face anthropometric survey of respirator users conducted by the National Institute of Occupational Safety and Heath (NIOSH),(3) this SOP details the 6 selected by the American Association of Textile Chemists & Colorists (AATCC) in their industry guidance document AATCC M14 2020 (4).

The tester was trained to perform the measurements quickly. Hand hygiene, surface disinfection, and use of face masks and a shield by the tester throughout, and a facemask by the participant except when absolutely necessary, mitigated risk.

Anthropometric instruments

We used a spreading caliper in keeping with methods used for the NIOSH bivariate fit panel. We also measured with flexible cord, and developed an instructional video so that participants could measure themselves safely at home (2).

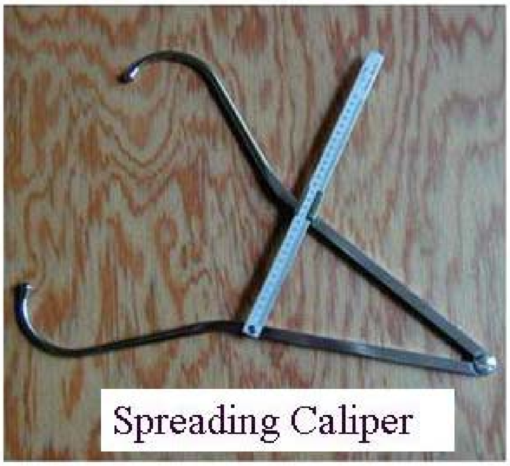

Landmark list

Selected with minor modifications from the Measurer's Handbook Appendix A.(3)

The Frankfort plane is with the participant at right angles to the tester, facing sideways. The tester is facing the participant's ear.

Menton: The inferior point of the mandible in the midsagittal plane (bottom of the chin).

Method: Subject stands with the head in the Frankfort Plane and the teeth together. Stand in front of the subject. Locate the landmark by palpation of the lower jawbone just under the chin, and place an adhesive dot on it.

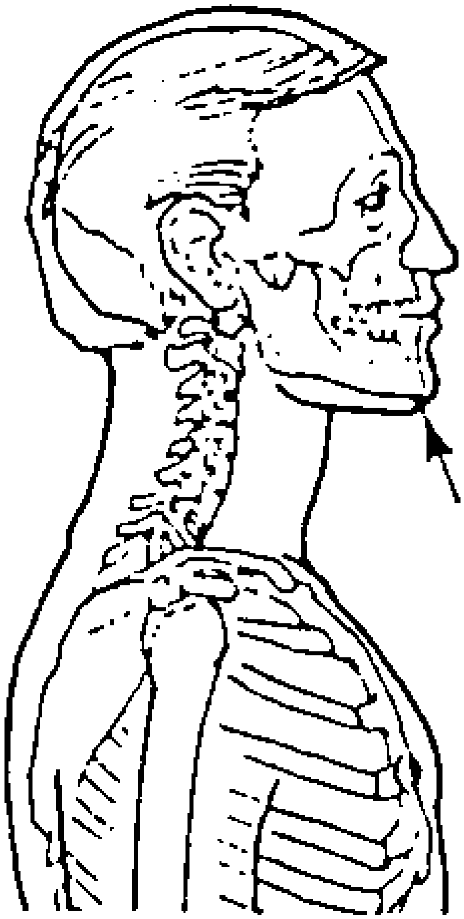

Sellion: The point of the deepest depression of the nasal bones at the top of the nose.

Method: The subject stands, looking straight ahead. Stand at the right of the subject and palpate the point of the deepest depression of the bridge of the nose in the midsagittal plane. On some subjects, however, there is no distinctly deepest point and judgment will have to be used to establish its location. Place an adhesive dot on the bridge of the nose at the landmark.

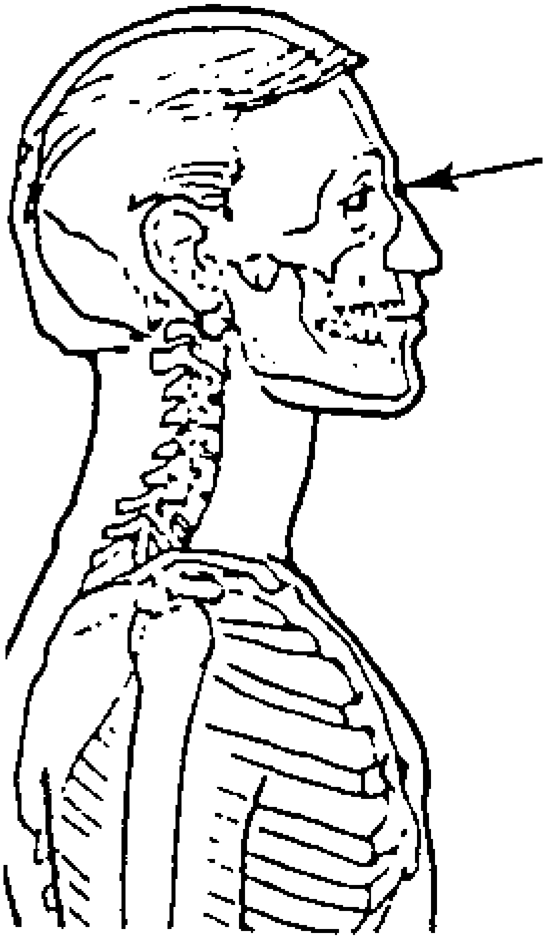

Zygion, right and left: The most lateral point on the zygomatic arch. (unmarked)

Method: The subject stands, looking straight ahead, with facial muscles relaxed. Stand in front of the subject and locate the most lateral point by palpation. (When unmarked, this is located by movement of the tips of the spreading caliper during measurement.) Place an adhesive dot on each landmark.

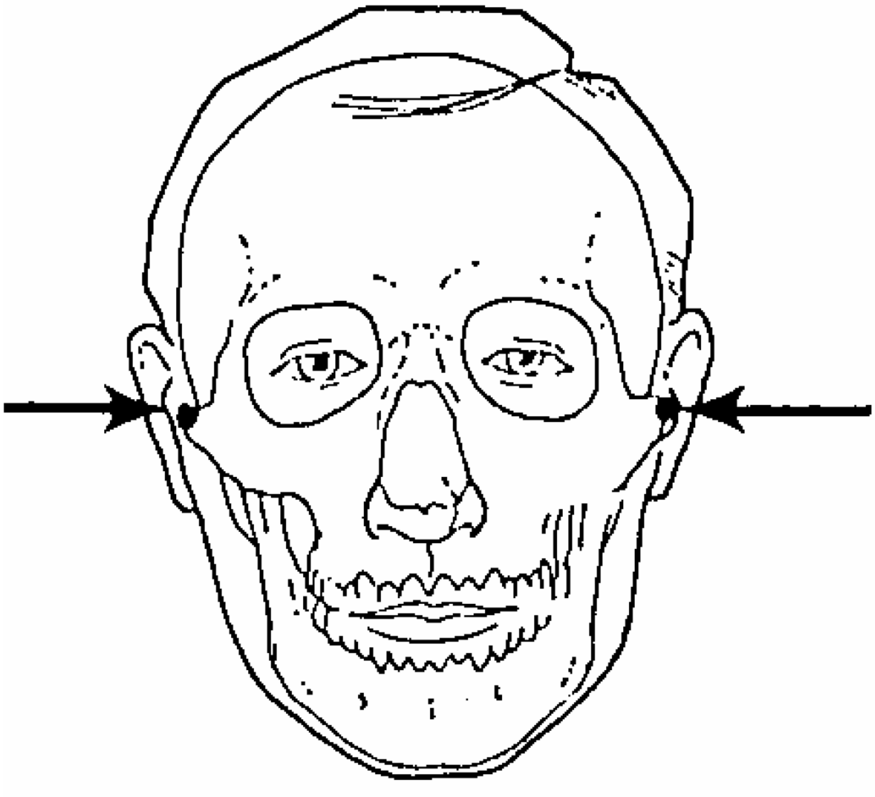

Dimension descriptions

BIZYGOMATIC BREADTH

The maximum horizontal breadth of the face between the zygomatic arches is measured with a spreading caliper. The subject sits looking straight ahead and with the teeth together (lightly occluded). Only enough pressure to ensure that the caliper tips are on the zygomatic arches is exerted.

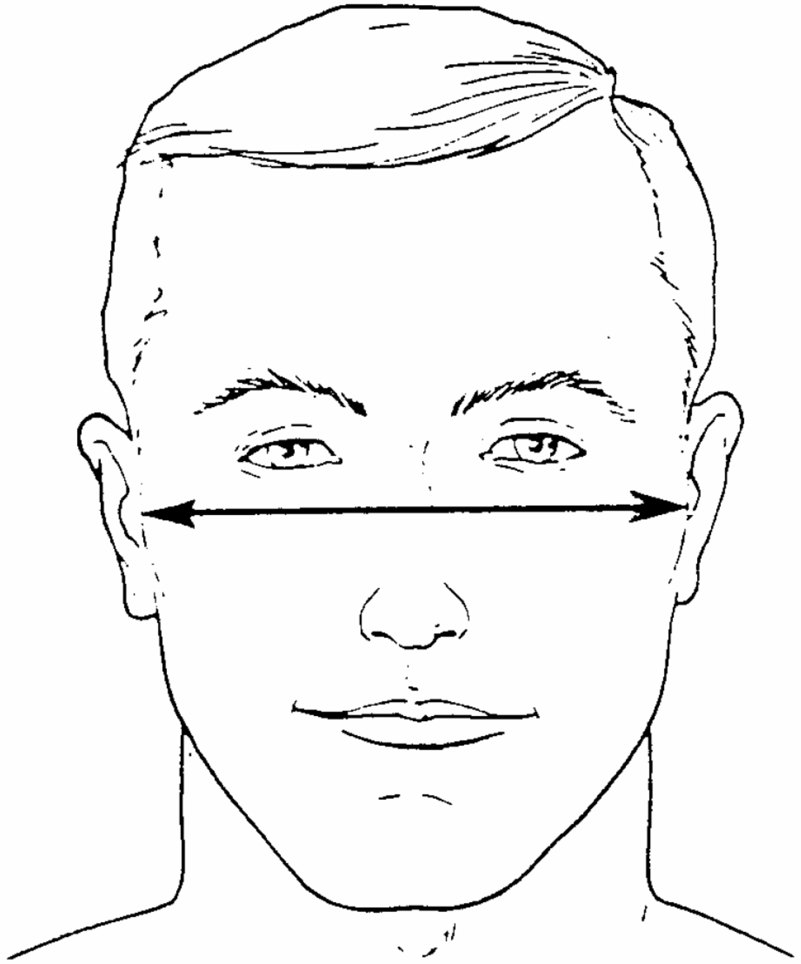

MENTON-SELLION LENGTH

The distance in the midsagittal plane between the Menton landmark at the bottom of the chin and the Sellion landmark at the deepest point of the nasal root depression is measured with a sliding caliper. The subject sits looking straight ahead and with the teeth together (lightly occluded). The fixed blade of the caliper is placed on Sellion. Only enough pressure to attain contact between the caliper and the skin is exerted.

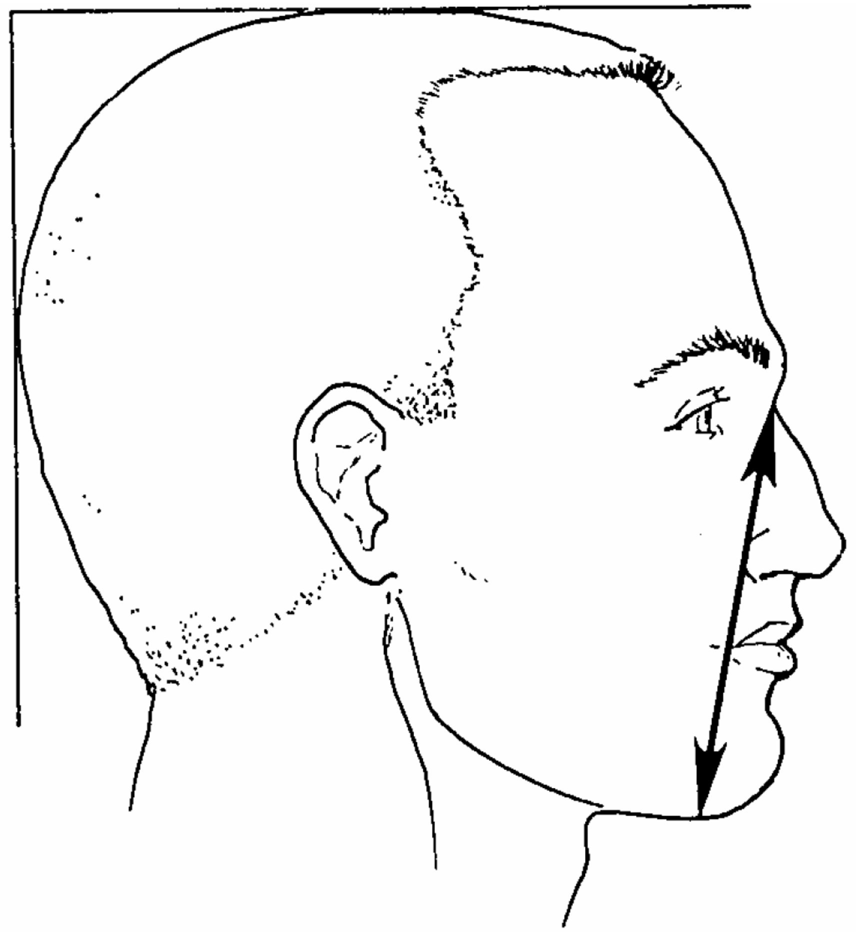

Operationalization for measurements in a pandemic

Post a large-print version (below) of these instructions where tester and participant can see it during testing. For the purposes of this study, because COVID was transmitting in our community throughout the study, we conducted only menton-sellion and bizygomatic measurements.

1. Maintain physical distance of at least 2 m except where directed by the protocol below. To facilitate this, use two moveable chairs at least that distance apart. Keep the tester's equipment in a work zone near the tester's chair.
2. Check that COVID-19 screening is clear for both participant and tester. Complete and comply with any workplace or research documentation required by the institution.
3. Participant and tester will be wearing masks under building policies. The participant's mask should be an earloop medical mask.
4. Participants who arrive in a cloth mask will be provided with a medical mask. They will change into the mask at > 4m distance from the tester.
5. The participant should not be wearing glasses or other eye protection. Contact lenses are not a problem.
6. Check that you have the required equipment
    a. Face shield, with paper or plastic bag labelled with tester's name
    b. Non-sterile gloves
    c. Hand sanitizer
    d. Anthropometric calipers
    e. Measuring tape
    f. Adhesive dots
    g. Disinfectant wipes suitable for surfaces (eg, chlorox or hydrogen peroxide)
    h. Your mobile phone with the Glasses On app installed, open to Camera>Video
    i. Stylus that allows you to interact with touch screen while wearing gloves
    j. Light-coloured standard-sized plastic membership card, such as a library card. Do not use a credit card or any other card which has visible personal information.
7. Put on the face shield.
8. Perform hand hygiene and ask the participant to do the same.
9. Put on non-sterile gloves.
10. Wipe down the calipers with a disinfectant wipe suitable for stethoscopes and medical equipment, or 70% ethanol.
11. Wipe down the flexible measuring tape using the same wipe.
12. Move your chair in.
13. Remove the gloves.
14. Perform hand hygeine
15. With the participant wearing a mask, locate and mark, using a small adhesive dot:
    a. Tragion bilaterally
    b. Gonion bilaterally
    c. Sellion.
16. Move your chair back.
17. Ask the participant to remove their mask.
18. Move your chair in, locate and mark:
    a. Zygion bilaterally

    b. Menton.
19. Move your chair back.
20. Turn on video or audio recording on your phone. Video itself is not needed, only the audio component of the recording. Put down the phone in your work zone.
21. Holding the caliper out to maximise the distance between you and the participant, but retaining fine motor control of its distal ends, measure in this order. Move your chair in as much as needed for these measurements. Speak the measurements aloud as you make them. Move quickly but carefully from each to the next.
    a. Bigonial
    b. Bizygomatic
    c. Menton-sellion
22. Put the calipers down and pick up the tape measure. Speak the measurements aloud as you make them. Measure:
    a. Bitragion-chin
    b. Bitragion-subnasale
23. Move your chair back.
24. Ask the participant to put on their mask and perform hand hygiene.
25. Put on disposable non-sterile gloves.
26. Wipe the calipers and tape with the disinfecting wipe.
27. Using the stylus, play back the video and record the dimensions on the case report form.
28. Wipe down the phone and the plastic card with a disinfecting wipe. Avoid getting liquid into ports.
29. Using the stylus, open the Glasses On app, select ‘pupillary distance’ and follow the prompts.
30. Explain the next steps to the participant. The facial recognition software does not work if the participant is wearing a mask. Show the participant how the app works if they have questions or are uncertain. Go through items 28-34 below. Tell them you will leave the room and that when they are finished they should place the phone face up in your work zone, put on their mask, perform hand hygiene, let you know that they are finished and sit down. They should also put on a mask to let you know if they have trouble.
31. Leave the room.
32. Participant removes mask.
33. Participant performs hand hygiene.
34. Participant performs interpupillary distance measurement, leaving the reading visible on the phone.
35. Participant puts down phone and plastic card in tester’s work zone.
36. Participant puts on mask, performs hand hygiene.
37. Participant lets tester know finished.
38. Participant resumes seat.
39. Record the interpupillary distance on the case report form.
40. Wipe down the phone and the plastic card with a disinfecting wipe.
41. Dispose of wipes.
42. Remove gloves and dispose of them.
43. Remove faceshield and place in the bag labelled with your name.
44. Perform hand hygiene.

F3407-20 – NIOSH Panel Based on Face Length and Width (5, 6)

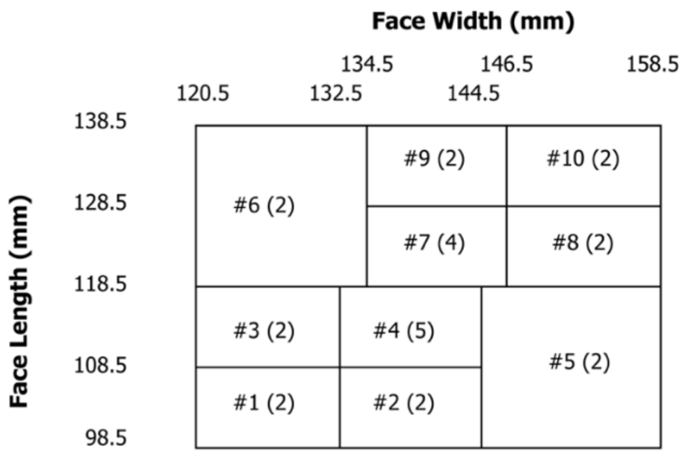

1. Clauser C, Tebbetts I, Bradtmiller B, McConville J, Gordon CC. Measurer's Handbook: U.S. Army Anthropometric Survey, 1987-1988. 1987.
2. Dulai R, Dulai G. Cord method of menton-sellion and bizygomatic measurement 2021 [Available from: https://www.youtube.com/channel/UCQVIQWOowNR7er6IHBGZySg.
3. Bradtmiller B, Friess M. A head-and-face anthropometric survey of U.S. respirator users. Final report. 2004 [Available from: https://www.nap.edu/resource/11815/Anthrotech_report.pdf.
4. AATCC. M14 Guidance and Considerations for General Purpose Textile Face Coverings: Adult 2020 [Available from: https://members.aatcc.org/store/m014/3085/.
5. ASTM International. ASTM F3407-21 Standard Test Method for Respirator Fit Capability for Negative-Pressure Half-Facepiece Particulate Respirators 2021 [Available from: https://www.astm.org/f3407-21.html.
6. TSI Incorporated. TSI Incorporated PORTACOUNT PRO+ 8038 Operation And Service Manual [Available from: https://www.manualslib.com/products/Tsi-Incorporated-Portacount-ProPlus-8038-6889864.html.
