# Supplementary material for: Measuring the fitted filtration efficiency of cloth masks, medical masks and respirators
Source: PLoS One. 2025 Apr 21;20(4):e0301310. doi: 10.1371/journal.pone.0301310 (PMC12011288; doi:10.1371/journal.pone.0301310)
Supplement: S2 Appendix — (PDF) [file pone.0301310.s012.pdf]

S2 Appendix

KN95, KF94 and non-certified mask purchase

We performed purposive sampling for KN95s, KN94s, and similar style masks. On 3 January 2022 we searched Amazon.ca for 'KN95' and separately for 'KN94' using the 'featured' sort order. We excluded masks that could not be delivered in the following three weeks, certified N95s, children's sizes, pleated masks, and duplicates from the same company as a previously chosen mask. We looked for the term 'KN95' or 'KF94' in text or in images of the masks or packaging. We did not require filtration data or a text assertion that the standard was met. We were unable to identify any KN95s that met the delivery criteria. We next searched for KN95s at (in order) Costco, Walmart, Shopper's Drug Mart, and found none were available. We used Masks4Canada's tipsheet (https://docs.google.com/document/d/1mapcgmkIzq4lva2xEIfNp7hgUlCyrpRWmUsF4dB62Z8/edit) in the order listed: from SteriPro we purchased GZ Harly KN95s (20 pack); these have been tested at the National Personal Protective Technology laboratory in the US (https://www.cdc.gov/niosh/npptl/respirators/testing/results/MTT-2020-95.1_International_GuangzhouHarley_L-103VKN95_TestReport_Redacted-508.pdf; GZ Harly KN95); other listed sellers of KN95s were sold out. Searching, in the order in which they were presented, sellers listed on Finder.com, we purchased KN95 Protective Face Masks (20 pack, sold by Goltum; Goltum KN95).

From the KN94 search we purchased: Flmaly K_F94 Disposable Face_Masks for Adults (50 pack, sold by kaixujf; listed as item 1; Glmaly KF94) and BuyEverything Disposable Face Masks, 3-Ply, Ear Loop (100 pack, sold by shaungyi30), listed as items 13-15 in the search; BuyEverything KF94). We also selected two masks that were similar in appearance but made no certification claims: the KEGIS Disposable 3D Face Mask (10 pack, sold by S&G Global; Kegis mask) which was the first listed 'Amazon Choice' in the KN95 search and the first listed 'bestseller' in the KF94 search, and the Chengde Technology Co 5-layer foldable face mask (10 pack, sold by CanaSafe Ltd; Chengde mask) which was the first mask in the KN95 search that was not a KN95 and not excluded. When the Chengde mask arrived, it was in packaging that included a KN95 claim, and it is included as a KN95 in the manuscript.

We excluded the following masks because the delivery dates were 27 Jan – 18 Feb 2022. In order, for the KN95 search the masks were: Huheta 5-Ply Breathable & Comfortable Filter Safety, Filter Efficiency≥95%, Protective Cup Dust Against PM2.5 (30 pack, sold by Penosule; listed as items 2 & 3 in the search) and SurigMac Powecom Black Face Mask (Authorized Respirator) GB2626-2006 (10 pack, sold by Penosule; listed as items 11 & 12 in the search).

N95s and CanN95s were available on this date from multiple sellers.
