# Supplementary material for: Measuring the fitted filtration efficiency of cloth masks, medical masks and respirators
Source: PLoS One. 2025 Apr 21;20(4):e0301310. doi: 10.1371/journal.pone.0301310 (PMC12011288; doi:10.1371/journal.pone.0301310)
Supplement: S3 Appendix — (PDF) [file pone.0301310.s013.pdf]

S3 Appendix

Rationale: filtration efficiency testing

Justification for use of all-particles mode rather than N95 mode

Between-study comparisons require attention to experimental detail. A similar experimental approach to ours, using the particle classifier within the Portacount (or equivalent), often called N95 mode, because it is used for testing respirator fit, tests charged particles of < 0.1 µm (1-3). This mode has the advantage of appearing rigorous and relevant: the SARS-CoV virus, for example, is about 0.1 µm in diameter (4). However, the rationale for this mode is that respirators contain electret materials that filter particularly *well* in this size range: the challenging size range (called the most penetrating particle) for respirator material is around 0.3 µm (5). This property makes the <0.1 µm classifier useful for fit testing: if particles of this size are detected inside the mask, it is inferred that they did not come through the material, but around the edge; if they exceed a threshold this is functionally important leak, and the respirator-participant combination is judged to have failed the fit test. We, and others (6), chose to test instead across the full range of submicron particles, as the more rigorous and more clinically-relevant test, which includes the most-penetrating-particle size. Virions do not exist in isolation but in respiratory particles of larger diameters (7). Though it is not known which particle sizes are most strongly associated with disease transmission, evidence that 3-5 µm particles may be most relevant (7-10) leads some investigators to include larger particle sizes and study the range 0.02 – 3 µm (11, 12) or 0.1 to 7 µm (13). Given the well-accepted U-shaped relationship between particle size and filtration with a nadir around 0.3 µm (14), our choice of particle range (0.02 – 1 µm) leads to estimates which are conservative compared with those obtained by testing only smaller particles or including much larger particles.

Detailed methods for testing

TSI's current model, the Portacount 8048, reports fit factors rounded or truncated to integers, which is of little consequence in the testing of respirators, where the expected fit is greater than 100. However, for testing lower-efficiency masks, the first decimal place is essential to precision and is provided by the older 8038 model. We randomized the order in which masks were tested using an online random number generator. We used the Demo-Training Mode database and created a new respirator on the device to use for testing, with a pass value of 1 and N95 not selected; the manufacturer, model, and style were arbitrary inputs as they have no impact on values reported by the device. We used the CSA Z94.4-2002 protocol to recover the fit factor of each mask for a series of seven exercises, corresponding to a total of 1.5 minutes required for the testing of each mask:

1. Normal breathing – Participants breathed as they normally would.
2. Deep breathing – Participants took deep breaths in and out.
3. Head turning – Participants repetitively turned their heads to face right and left. We guided them to spend about three seconds facing each way and, in each transition (i.e., spend three seconds facing left, then three seconds to transition from facing left to right, then spend three seconds facing right before transitioning again).

4. Head nodding – Participants nodded their heads up and down. Similar to the head turning exercise, we guided them to spend about three seconds in each nod direction and three seconds in each transition.
5. Talking out loud – Participants read the Rainbow Passage, a phonetically balanced standardized script that imitates the sounds and mouth movements of typical English speech.
6. Bending over – Participants stood up and repeatedly tilted their upper body forward and then back to standing. Like exercises 3 and 4, we guided participants to spend about three seconds transitioning and three seconds at each movement checkpoint (i.e., standing up straight and bent forward at the hips). This is the only exercise in which participants were not seated.
7. Normal Breathing – Participants breathed as they normally would.

For each activity, the Portacount cycles between drawing air from ambient and from the mask, measuring particles using a condensation counter. When used according to CSA Z94.4-2002 protocol the Portacount measures in 'all particles' mode it detects particles in the size range 0.02 to 1 µm. We ensured that participants did not have on eyewear during the exercises because it could have altered the intended fit of the mask. Fit factor for each exercise is a dimensionless number, calculated as the ratio of ambient to mask particles for that exercise; eg, a fit factor of 2 reflects twice the particles outside compared with inside. Overall fit factor for each mask was calculated using built-in software, as the geometric mean of the fit factor for each exercise. We converted all fit factor values, defined as the ratio of particle concentration outside the mask to inside the mask, to fitted filtration efficiency using 1-(1/fit factor)×100. Fitted filtration efficiency describes the percentage of particles filtered by the mask; eg, a fit factor of 2 corresponds to fitted filtration efficiency of 50%.

1. Davies A, Thompson KA, Giri K, Kafatos G, Walker J, Bennett A. Testing the efficacy of homemade masks: would they protect in an influenza pandemic? Disaster medicine and public health preparedness. 2013;7(4):413-8.
2. O'Kelly E, Arora A, Pirog S, Ward J, Clarkson PJ. Comparing the fit of N95, KN95, surgical, and cloth face masks and assessing the accuracy of fit checking. PloS one. 2021;16(1):e0245688.
3. Blachere FM, Lemons AR, Coyle JP, Derk RC, Lindsley WG, Beezhold DH, et al. Face mask fit modifications that improve source control performance. American journal of infection control. 2022;50(2):133-40.
4. Varga Z, Flammer AJ, Steiger P, Haberecker M, Andermatt R, Zinkernagel A, et al. Electron microscopy of SARS-CoV-2: a challenging task - Authors' reply. Lancet. 2020;395(10238):e100.
5. He X, Reponen T, McKay RT, Grinshpun SA. Effect of Particle Size on the Performance of an N95 Filtering Facepiece Respirator and a Surgical Mask at Various Breathing Conditions. Aerosol Sci Technol. 2013;47(11):1180-7.
6. Reponen T, Lee SA, Grinshpun SA, Johnson E, McKay R. Effect of fit testing on the protection offered by n95 filtering facepiece respirators against fine particles in a laboratory setting. The Annals of occupational hygiene. 2011;55(3):264-71.
7. Anand S, Mayya YS. Size distribution of virus laden droplets from expiratory ejecta of infected subjects. Sci Rep. 2020;10(1):21174.
8. Fennelly KP. Particle sizes of infectious aerosols: implications for infection control. The Lancet Respiratory medicine. 2020;8(9):914-24.
9. Alsved M, Nygren D, Thuresson S, Fraenkel C-J, Medstrand P, Löndahl J. Size distribution of exhaled aerosol particles containing SARS-CoV-2 RNA. Infectious Diseases. 2022:1-6.
10. Whyte HE, Montigaud Y, Audoux E, Verhoeven P, Prier A, Leclerc L, et al. Comparison of bacterial filtration efficiency vs. particle filtration efficiency to assess the performance of non-medical face masks. Sci Rep. 2022;12(1):1188.
11. Sickbert-Bennett EE, Samet JM, Clapp PW, Chen H, Berntsen J, Zeman KL, et al. Filtration Efficiency of Hospital Face Mask Alternatives Available for Use During the COVID-19 Pandemic. JAMA internal medicine. 2020;180(12):1607-12.
12. Clapp PW, Sickbert-Bennett EE, Samet JM, Berntsen J, Zeman KL, Anderson DJ, et al. Evaluation of Cloth Masks and Modified Procedure Masks as Personal Protective Equipment for the Public During the COVID-19 Pandemic. JAMA internal medicine. 2020.
13. Brooks JT, Beezhold DH, Noti JD, Coyle JP, Derk RC, Blachere FM, et al. Maximizing Fit for Cloth and Medical Procedure Masks to Improve Performance and Reduce SARS-CoV-2 Transmission and Exposure, 2021. MMWR Morbidity and mortality weekly report. 2021;70(7):254-7.
14. Zangmeister CD, Radney JG, Vicenzi EP, Weaver JL. Filtration Efficiencies of Nanoscale Aerosol by Cloth Mask Materials Used to Slow the Spread of SARS-CoV-2. ACS Nano. 2020;14(7):9188-200.
