# Supplementary material for: Measuring the fitted filtration efficiency of cloth masks, medical masks and respirators
Source: PLoS One. 2025 Apr 21;20(4):e0301310. doi: 10.1371/journal.pone.0301310 (PMC12011288; doi:10.1371/journal.pone.0301310)
Supplement: S4 Appendix — (PDF) [file pone.0301310.s014.pdf]

S4 Appendix

Qualitative Data Collection

We assessed qualitative mask performance by asking each participant to rate glasses fog caused by the mask, discomfort of the mask, and mask leakage, using standardized 7-point Likert scales with anchors. For glasses fog testing, we invited participants to wear their own eyewear, or wear provided safety glasses. We asked participants to give their best attempt to fog up the glasses. To assess mask leak, we asked participants to place their fingers at mask leakage points, ie, under the eyes, cheeks and chin, then forcibly exhale. In addition to these ratings, we asked participants if they experienced a series of six pre-defined common issues rated to mask fit. We also recorded as free text any comments made by participants about mask comfort or fit.

Glasses Fog Rating Scale

1. I cannot induce fogging with any amount of effort
2. A flicker of fog gone instantly
3.
4. Moderate fog halfway up the lens, transient
5.
6. Severe: lots of fog all the way up the lens but persists for less than a count of three seconds
7. Very severe: lots of fog all the way up the lens and persists for a count of three seconds or more

We did not create anchors for '3' and '5'; they are midway between the defined anchors.

Leak Rating Scale

1. I do not detect any leaks and the seal to my face feels secure. If I have experience in wearing N95s, the mask feels comparable to an N95
2. I do not detect any leaks but the seal to my face is not perfectly secure
3. I detect minor leaks in one area
4. I detect minor leaks in more than one area
5. I detect at least one major leak
6. I detect severe leaking
7. The mask is poorly secured at the edges and makes poor contact with the face

Discomfort Rating Scale

1. Extremely comfortable: I hardly know I have it on. There are no uncomfortable pressure points. My nose feels clear. The mask doesn't move with movement or talking and feels secure
2. Very comfortable: I feel like I could wear this for hours. There are no uncomfortable pressure points. My nose feels clear. The mask may move a little with movement or talking but it still feels secure
3. Comfortable
4. Quite comfortable: there are pressure points, or my nose feels stuffy, or there is unacceptable movement
5. Uncomfortable
6. Very uncomfortable: there are unacceptable pressure points, or my nose is extremely blocked, or there is a lot of movement to the point that I feel I should be readjusting it
7. Extremely uncomfortable: I feel like I have to take this off immediately
