# Supplementary material for: Measuring the fitted filtration efficiency of cloth masks, medical masks and respirators
Source: PLoS One. 2025 Apr 21;20(4):e0301310. doi: 10.1371/journal.pone.0301310 (PMC12011288; doi:10.1371/journal.pone.0301310)
Supplement: S5 Appendix — (PDF) [file pone.0301310.s015.pdf]

S5 Appendix

Cloth Mask Knowledge Exchange

In 2020, researchers from the Centre of Excellence in Protective Equipment and Materials (CEPEM) at McMaster University formed a coalition with colleges, mask makers (business, altruistic, and artisanal), fabric distributors, standards experts, and a technology transfer centre. Research participants were included. Through bi-weekly meetings of an executive group, this coalition reviewed these data and data from other, ongoing filtration studies at CEPEM and advised the research team about direction.

Co-chairs

Darren Lawless, assistant vice-president of research, innovation and partnerships, McMaster University

Catherine Clase, professor of medicine, McMaster University

Executive

Marilyn McNeil-Morin, director at Fashion Exchange, George Brown College

Jennifer Wright, professor of fashion design, Fanshawe College

Cassie Hopper, professor, Fanshawe College

Helen Brunet, strategic development manager, Vestechpro technology transfer centre

Evelina Agostini, Veratex Lining Inc and Zinman Inc

Barry Diamond, managing director, Veratex Lining Inc

Joe Camillo, owner, Niko Apparel Inc

Barb Round, quilter; liaison for Quilters Canada

Joan Fearnley, choral director and mask designer, liaison for Choral Canada

Susan Bartlett, professor of medicine, McGill University

Rebecca Rudman, co-lead, Windsor-Essex Sewing Force

Haridoss Sarma, PhD, engineer and standards expert

Scott Laengert, PhD candidate, McMaster University

Amanda Tomkins, electrical and biomedical engineering year 4, McMaster University

Ranmeet Dulai, health sciences year 4, McMaster University

Gurleen Dulai, chemical and biomedical engineering year 4, McMaster University

Sarah Rassenberg, chemical engineering year 4, McMaster University

Members

Ching Mark, designer, Niko Apparel Inc

Maya Goldenberg, professor of philosophy, University of Guelph

Marian Reich, patient partner

Isabelle Lavallee, Veratex Lining Inc

Ravi Selvaganapathy, director, Centre of Excellence in Protective Equipment and Materials

Charles de Lannoy, assistant professor of chemical engineering, McMaster University

David Latulippe, associate professor of chemical engineering, McMaster University

Benzhong (Robin) Zhao, assistant professor of civil engineering, McMaster University
