# Supplementary material for: Measuring the fitted filtration efficiency of cloth masks, medical masks and respirators
Source: PLoS One. 2025 Apr 21;20(4):e0301310. doi: 10.1371/journal.pone.0301310 (PMC12011288; doi:10.1371/journal.pone.0301310)
Supplement: S6 Appendix — (PDF) [file pone.0301310.s016.pdf]

S6 Appendix

Limitations

1. Limited sample of masks
2. Participant sample size limited by respondent burden and at times by pandemic-related restrictions on in-person research.
3. One design of cloth masks tested, commercially available masks not sampled
4. Cloth mask did not contain a spunbond polypropylene layer
5. ASTM barrier face covering standard F3407-21 tests masks edge-sealed to a plate; data not provided (1)
6. No source control data, either at rest, with exertion, coughing or sneezing
7. No information on washability of masks or sterilization of respirators (2-4)
8. Comfort assessed after wearing for minutes, not hours
9. No information on reusability or extended use of masks intended to be disposable
10. No information on the effects of humidity on filtration
11. No information on relationship of filtration to respiratory volume (minute ventilation)(5)
12. We included patients with facial hair only in the study of mask hacks, because with N = 10 we judged it good to be inclusive; 3 participants had short beards (6) For the mask types and overmasking studies with fewer participants, we eliminated this effect by excluding people with beards (7)
13. No adjustment for multiple comparisons
14. A nosewire was not included in the cloth masks because of concerns about breakage, safety, and to maximize longevity when washed with clothes. The mask is designed so that when cinched on elastic or ties, it forms a neat curve that fits to the wearers' face (S1 Fig). Findings from this mask may not generalize to other less well-constructed masks. The filtration of this mask might be improved by the addition of a nosewire, as has been previously reported for a different mask (8). For overmasking, the nosewire in the medical mask likely reduces leak in this area and there may be no impact of the absence of a nosewire when used as an overmask.

1. ASTM International. ASTM F3407-21 Standard Test Method for Respirator Fit Capability for Negative-Pressure Half-Facepiece Particulate Respirators 2021 [Available from: https://www.astm.org/f3407-21.html.
2. Kumar A, Sangeetha DN, Yuvaraj R, Menaka M, Subramanian V, Venkatraman B. Quantitative Performance Analysis of Respiratory Facemasks Using Atmospheric and Laboratory Generated Aerosols Following with Gamma Sterilization. Aerosol and Air Quality Research. 2021;21(1):200349.
3. Kumar A, Bhattacharjee B, Sangeetha DN, Subramanian V, Venkatraman B. Evaluation of filtration effectiveness of various types of facemasks following with different sterilization methods. Journal of Industrial Textiles. 2021;51(2_suppl):3430S-65S.

4. Kumar A, Joshi S, Venkatesan S, Balasubramanian V. A detailed investigation of N95 respirator sterilization with dry heat, hydrogen peroxide, and ionizing radiation. Journal of Industrial Textiles. 2022;51(1_suppl):378S-405S.
5. Li X, Ding P, Deng F, Mao Y, Zhou L, Ding C, et al. Wearing time and respiratory volume affect the filtration efficiency of masks against aerosols at different sizes. Environ Technol Innov. 2022;25:102165.
6. CDC Centers for Disease Control and Prevention. To Beard or not to Beard? That's a good Question! 2017 [Available from: https://blogs.cdc.gov/niosh-science-blog/2017/11/02/noshave/.
7. Prince SE, Chen H, Tong H, Berntsen J, Masood S, Zeman KL, et al. Assessing the effect of beard hair lengths on face masks used as personal protective equipment during the COVID-19 pandemic. Journal of exposure science & environmental epidemiology. 2021;31(6):953-60.
8. Clapp PW, Sickbert-Bennett EE, Samet JM, Berntsen J, Zeman KL, Anderson DJ, et al. Evaluation of Cloth Masks and Modified Procedure Masks as Personal Protective Equipment for the Public During the COVID-19 Pandemic. JAMA internal medicine. 2020.
