# Supplementary material for: Measuring the fitted filtration efficiency of cloth masks, medical masks and respirators
Source: PLoS One. 2025 Apr 21;20(4):e0301310. doi: 10.1371/journal.pone.0301310 (PMC12011288; doi:10.1371/journal.pone.0301310)
Supplement: Attachment — Submitted filename: Response to questions 2025-02-03.docx [file pone.0301310.s019.docx]

**Response to questions**

1. Please note that your Data Availability Statement is currently missing [the repository name]. If your manuscript is accepted for publication, you will be asked to provide these details on a very short timeline. We therefore suggest that you provide this information now, though we will not hold up the peer review process if you are unable.

Previously:

Results

The 12 participants were 21 – 55 years, 58% female, 25% non-European, NIOSH 1 to 10 (Table and S1 Table). Data are shared in an online repository at https://osf.io/58g2j/.

Revised to:

Results

The 12 participants were 21 – 55 years, 58% female, 25% non-European, NIOSH 1 to 10 (Table and S1 Table). Data are shared in an online repository, OSFHOME, at https://osf.io/58g2j/.

2. Please include your tables as part of your main manuscript and remove the individual files. Please note that supplementary tables (should remain/ be uploaded) as separate "Supporting Information" files

Thank you for confirming by email that this was already done.

3. "We note that there is identifying data in the Supporting Information file < S1_Table.pdf >. Due to the inclusion of these potentially identifying data, we have removed this file from your file inventory. Prior to sharing human research participant data, authors should consult with an ethics committee to ensure data are shared in accordance with participant consent and all applicable local laws.

We have changed the ages of the participants to age ranges, as requested. Thank you for this suggestion.

Best wishes and thanks,

Catherine
