# Supplementary material for: Measuring the fitted filtration efficiency of cloth masks, medical masks and respirators
Source: PLoS One. 2025 Apr 21;20(4):e0301310. doi: 10.1371/journal.pone.0301310 (PMC12011288; doi:10.1371/journal.pone.0301310)
Supplement: Attachment — Submitted filename: Response to reviews 2024-07-14.docx [file pone.0301310.s018.docx]

Response to reviews 2024-06-26

PONE-D-24-09859

Measuring the fitted filtration efficiency of cloth masks, medical masks and respirators

PLOS ONE

Dear Dr. Clase,

Thank you for submitting your manuscript to PLOS ONE. After careful consideration, we feel that it has merit but does not fully meet PLOS ONE’s publication criteria as it currently stands. Therefore, we invite you to submit a revised version of the manuscript that addresses the points raised during the review process.

==============================

The work is interested, however, reviewers have given comments for improvement. E.g. It should be mentioned that FE is based on total measured particle count and size-dependent.

==============================

Please submit your revised manuscript by Jul 15 2024 11:59PM. If you will need more time than this to complete your revisions, please reply to this message or contact the journal office at plosone@plos.org. When you're ready to submit your revision, log on to https://www.editorialmanager.com/pone/ and select the 'Submissions Needing Revision' folder to locate your manuscript file.

Please include the following items when submitting your revised manuscript:

•	A rebuttal letter that responds to each point raised by the academic editor and reviewer(s). You should upload this letter as a separate file labeled 'Response to Reviewers'.

•	A marked-up copy of your manuscript that highlights changes made to the original version. You should upload this as a separate file labeled 'Revised Manuscript with Track Changes'.

•	An unmarked version of your revised paper without tracked changes. You should upload this as a separate file labeled 'Manuscript'.

If you would like to make changes to your financial disclosure, please include your updated statement in your cover letter. Guidelines for resubmitting your figure files are available below the reviewer comments at the end of this letter.

If applicable, we recommend that you deposit your laboratory protocols in protocols.io to enhance the reproducibility of your results. Protocols.io assigns your protocol its own identifier (DOI) so that it can be cited independently in the future. For instructions see: https://journals.plos.org/plosone/s/submission-guidelines#loc-laboratory-protocols. Additionally, PLOS ONE offers an option for publishing peer-reviewed Lab Protocol articles, which describe protocols hosted on protocols.io. Read more information on sharing protocols at https://plos.org/protocols?utm_medium=editorial-email&utm_source=authorletters&utm_campaign=protocols.

We look forward to receiving your revised manuscript.

Kind regards,

Yasir Nawab, PhD

Academic Editor

PLOS ONE

Journal requirements:

When submitting your revision, we need you to address these additional requirements.

1. Please ensure that your manuscript meets PLOS ONE's style requirements, including those for file naming. The PLOS ONE style templates can be found at

https://journals.plos.org/plosone/s/file?id=wjVg/PLOSOne_formatting_sample_main_body.pdf and

<https://journals.plos.org/plosone/s/file?id=ba62/PLOSOne_formatting_sample_title_authors_affiliations.pdf>

Done, many thanks for providing those templates.

2. Thank you for stating the following in the Competing Interests section: "Amanda Tomkins is a member of Dr Qiyin Fang’s research group which worked on the silicone mask brace. She is also a member of the cloth mask knowledge exchange, a stakeholder group that includes cloth mask manufacturers and fabric distributors.

Catherine Clase has received consultation, advisory-board membership, honoraria, or research funding from the Ontario Ministry of Health, Sanofi, Pfizer, Leo Pharma, Astellas, Janssen, Amgen, Boehringer-Ingelheim, Baxter and, through LiV Academy, AstraZeneca. In 2018 she co-chaired a KDIGO potassium controversies conference sponsored at arm's length by Fresenius Medical Care, AstraZeneca, Vifor Fresenius Medical Care, Relypsa, Bayer HealthCare and Boehringer Ingelheim. She co-chairs the cloth mask knowledge exchange, a stakeholder group that includes cloth mask manufacturers and fabric distributors. She is editor-in-chief of MaskEvidence.org.

Ken G Drouillard is a member of the WE-SPARK Health Institute, University of Windsor and receives funding from the Natural Sciences and Engineering Research Council of Canada, Environment and Climate Change Canada and Ontario Ministry of Conservation, Environment and Parks. In 2020-2022 he acted as science consultant to the Windsor-Essex Sewing Force, a community group engaged in the design, sewing and donation of cloth masks to healthcare providers and vulnerable populations of Southern Ontario. He is a member of the cloth mask knowledge exchange.

Charles-Francois de Lannoy has received funding from various branches of The Natural Sciences and Engineering Research Council of Canada (NSERC), Ontario Centre of Innovation (OCI), formerly Ontario Centres of Excellence (OCE), Ontario Water Consortium (OWC) formerly Southern Ontario Water Consortium (SOWC), Canada First Research Excellence Fund (CFREF), Ontario Together Fund, and Federal Economic Development Agency for Southern Ontario (FedDev). He is a member of cloth mask knowledge exchange, a stakeholder group that includes cloth mask manufacturers and fabric distributors.

Darren Lawless co-chairs the cloth mask knowledge exchange, and all authors are members.

Other authors have no additional disclosures.

"

Reviewed, unchanged.

Please confirm that this does not alter your adherence to all PLOS ONE policies on sharing data and materials, by including the following statement: ""This does not alter our adherence to PLOS ONE policies on sharing data and materials.” (as detailed online in our guide for authors http://journals.plos.org/plosone/s/competing-interests). If there are restrictions on sharing of data and/or materials, please state these. Please note that we cannot proceed with consideration of your article until this information has been declared.

We added a sentence on data sharing to Results, paragraph 1:

The 12 participants were 21 – 55 years, 58% female, 25% non-European, NIOSH 1 to 10 (table and supplementary table 1). Data are shared in an online repository at <https://osf.io/58g2j/>.

We added the declaration above at the end of the Acknowledgements, please let us know if this wasn’t correct.

This does not alter our adherence to PLOS ONE policies on sharing data and materials.

Please include your updated Competing Interests statement in your cover letter; we will change the online submission form on your behalf.

No updates needed

3. We note that you have indicated that there are restrictions to data sharing for this study. PLOS only allows data to be available upon request if there are legal or ethical restrictions on sharing data publicly. For more information on unacceptable data access restrictions, please see http://journals.plos.org/plosone/s/data-availability#loc-unacceptable-data-access-restrictions.

Before we proceed with your manuscript, please address the following prompts:

a) If there are ethical or legal restrictions on sharing a de-identified data set, please explain them in detail (e.g., data contain potentially identifying or sensitive patient information, data are owned by a third-party organization, etc.) and who has imposed them (e.g., a Research Ethics Committee or Institutional Review Board, etc.). Please also provide contact information for a data access committee, ethics committee, or other institutional body to which data requests may be sent.

b) If there are no restrictions, please upload the minimal anonymized data set necessary to replicate your study findings to a stable, public repository and provide us with the relevant URLs, DOIs, or accession numbers. For a list of recommended repositories, please see

https://journals.plos.org/plosone/s/recommended-repositories. You also have the option of uploading the data as Supporting Information files, but we would recommend depositing data directly to a data repository if possible.

We will update your Data Availability statement on your behalf to reflect the information you provide.

Thank you for this. We have uploaded the data to https://osf.io/58g2j/

4. Please include captions for your Supporting Information files at the end of your manuscript, and update any in-text citations to match accordingly. Please see our Supporting Information guidelines for more information: http://journals.plos.org/plosone/s/supporting-information.

Done, thanks for the detailed instructions

[Note: HTML markup is below. Please do not edit.]

Reviewers' comments:

Reviewer's Responses to Questions

Comments to the Author

1. Is the manuscript technically sound, and do the data support the conclusions?

The manuscript must describe a technically sound piece of scientific research with data that supports the conclusions. Experiments must have been conducted rigorously, with appropriate controls, replication, and sample sizes. The conclusions must be drawn appropriately based on the data presented.

Reviewer #1: Yes

Reviewer #2: Yes

________________________________________

2. Has the statistical analysis been performed appropriately and rigorously?

Reviewer #1: Yes

Reviewer #2: Yes

________________________________________

3. Have the authors made all data underlying the findings in their manuscript fully available?

The PLOS Data policy requires authors to make all data underlying the findings described in their manuscript fully available without restriction, with rare exception (please refer to the Data Availability Statement in the manuscript PDF file). The data should be provided as part of the manuscript or its supporting information, or deposited to a public repository. For example, in addition to summary statistics, the data points behind means, medians and variance measures should be available. If there are restrictions on publicly sharing data—e.g. participant privacy or use of data from a third party—those must be specified.

Reviewer #1: Yes

Reviewer #2: Yes

________________________________________

4. Is the manuscript presented in an intelligible fashion and written in standard English?

PLOS ONE does not copyedit accepted manuscripts, so the language in submitted articles must be clear, correct, and unambiguous. Any typographical or grammatical errors should be corrected at revision, so please note any specific errors here.

Reviewer #1: Yes

Reviewer #2: Yes

________________________________________

5. Review Comments to the Author

Please use the space provided to explain your answers to the questions above. You may also include additional comments for the author, including concerns about dual publication, research ethics, or publication ethics. (Please upload your review as an attachment if it exceeds 20,000 characters)

Reviewer #1: This manuscript excellently explains the experimental conduct by the authors.

I would recommend to accept it in current condition. Just minor revision to english proficiency.

Thanks to this reviewer for their very generous comments and positive recommendations.

The manuscript is good to accept.

Reviewer #2: Comments to the Author

Measuring the fitted filtration efficiency (FFE) of cloth masks, medical masks, and respirators for small aerosol (0.02 – 1 µm) and effect on FFE by user modifications, over masking etc. The study is quite interesting and explained well. However, the manuscript needs to be revised before consideration. The section-wise comments/suggestions on the article are given below:

Abstract:

Closed room with ambient particles supplemented with salt particles. It would be better if a total fraction of ambient and salt aerosol were given.

Absolutely, we agree. Another better approach is to conduct the experiments in a sealed chamber, so that only salt particles are in play. The equipment to measure the particle types and sizes, and a sealed chamber were not available at our centre. We think that ambient particles are likely a good surrogate for bioaerosol, and salt particles likely lead to underestimation of filtration, compared with less-dense bioaerosols, so that the results we’ve obtained for filtration are conservative.

**We’ve added this issue as a limitation to the Discussion:**

We recognize additional limitations (S6 Appendix). Filtration properties vary with the size and density of particles and it is a limitation that we have no information on this given that our methodology could not delineate aerosols present within the test space or interior of the mask contributed by ambient particles from NaCl particles produced by the particle generator. It is possible that the proportion of NaCl and ambient particles varied on different days. However, we note that our methodology is broadly consistent with multiple studies using NaCl particle generators and TSI PortaCount Respirator Fit testers to measure fit factors and mask performance (5, 45, 54, 57, 59).

The FFE need to be written with a standard deviation like FFE ± SD.

Done, throughout

Introduction:

The introduction is written shortly and concisely; however, some recent literature was missing in a similar domain, which can also be included in the main manuscript or supplementary file. A few are given below:

• Quantitative performance analysis of respiratory facemasks using atmospheric and laboratory generated aerosols following with gamma sterilization. Aerosol and Air Quality Research, 21(1), 200349.

• Evaluation of filtration effectiveness of various types of facemasks following with different sterilization methods. Journal of Industrial Textiles, 51(2_suppl), 3430S-3465S.

• A detailed investigation of N95 respirator sterilization with dry heat, hydrogen peroxide, and ionizing radiation. Journal of Industrial Textiles, 51(1_suppl), 378S-405S.

**We added the issue of sterilization, and these references, to S6 Appendix:**

7.	No information on washability of masks or sterilization of respirators (2-4)

Methods:

Page 5: How long does a generated NaCl aerosol drying in a room environment? The room temperature and humidity also play a role. The temperature and humidity may be given in the manuscript.

Aerosols of this size dry in milliseconds in a room environment. **We added this sentence to the discussion:**

(Salt particles are generated as saline but dehydrate to solid salt within milliseconds (71, 74).

The particle counts between 2000 to 20,000 in per cm3 or per litter or per m3.

Our apologies, we have added the units. **This sentence now reads:**

We proceeded with testing if the total particle count was between 2000 and 20,000 per cm^2^.

Page 6: The statistical methods section needs to be revised into simple sentences. Entire paragraph is given in one sentence. It is tough to understand.

Sorry about that. We have broken this up, **it now reads:**

We used SYSTAT v13, Inpixon, Palo Alto, CA for statistical analyses. We analyzed the transformed variable filtration efficiency. We used parametric tests (ANOVA with post hoc Tukey’s honestly significant difference) when data met normality assumptions by Lilliefors test. We used non-parametric tests (Kruskal-Wallis with post-hoc Conover-Inman pairwise comparisons) when they did not, estimating mean and 95% confidence intervals or median and interquartile range, respectively. Throughout, we regarded p<0.05 as statistically significant.

Results:

Table 1: mean and SD are given as comma-separated; it should be better if it is given FE ± SD.

Done.

Supplementary file Fig. 1: A two-layer pleated mask should have pleated counts in each mask type. Levels 1, and 3 and certified masks should have detailed specifications like material density, fibre diameter, nose clip, breathing resistance etc.

We have added the pleat numbers and a webreference for the detailed pattern for the cloth mask. We have added information about the nosewires and the physical structure of the commercial masks. We are sorry, we do not have information on breathing resistance or fibre diameters.

Supplementary file page19: “Given the well-accepted U-shaped relationship between particle size and filtration with a nadir around 0.3 µm,23 it seems probable that our choice of particle range (0.02 – 1 µm) leads to estimates that are conservative compared with those obtained by testing only smaller particles or including much larger particles.”

Here the sentence should be modified because many studies have been conducted for particle ranges from 10 nm to 10 µm.

Thanks for this, that is helpful. The shape of the curves are well know, as the reviewer observes. We have changed this (in **S3 Appendix**) to reflect the lack of uncertainty on this point.

Given the well-accepted U-shaped relationship between particle size and filtration with a nadir around 0.3 µm (1), our choice of particle range (0.02 – 1 µm) leads to estimates which are conservative compared with those obtained by testing only smaller particles or including much larger particles.

Supplementary file page 22: In the Glass fog rating scale, rating numbers 3 & 5 are blank. Something missing?

Thanks for pointing this out, we can see that that might be puzzling. Scales like this don’t have to have every point defined. We’ve clarified this by adding the text in **S4 Appendix**:

We did not create anchors for ‘3’ and ‘5’; they are midway between the defined anchors.

Supplementary file page 36: Limitation and area for future research: many works have been already performed for many tabulated points. However, agreed with the authors, many areas need to be further researched.

We acknowledge many informative papers available on many of these topics. We thought a detailed review of the literature on all these points beyond the scope of this work, and accordingly changed the heading, in **S6 Appendix** from “Limitations and areas for future research” to “Limitations”.

Discussion:

Page 10: Previous studies of FFE for medical and non-medical masks have been limited by minimal sample size and incomplete description of the mask. Please see each detail given for FE and mask characterization in the study below. Hence the sentence may be modified accordingly.

Thank you for this important point; we are discussing fitted filtration efficiency on humans, which has specific challenges, particularly for work conducted during a pandemic. **We have changed the sentence in the Discussion to reflect this**:

Previous studies of fitted filtration efficiency for medical and non-medical masks have been limited by very small sample sizes and incomplete descriptions of the masks.

**to now read:**

Many previous studies of fitted filtration efficiency with human participants, for medical and non-medical masks, have been limited by very small sample sizes or incomplete descriptions of the masks.

• Quantitative performance analysis of respiratory facemasks using atmospheric and laboratory generated aerosols following with gamma sterilization. Aerosol and Air Quality Research, 21(1), 200349.

• Evaluation of filtration effectiveness of various types of facemasks following with different sterilization methods. Journal of Industrial Textiles, 51(2_suppl), 3430S-3465S.

• A detailed investigation of N95 respirator sterilization with dry heat, hydrogen peroxide, and ionizing radiation. Journal of Industrial Textiles, 51(1_suppl), 378S-405S.

Thank you for these interesting references, they have been added to S6 Appendix

Page 11: The filtration efficiency should also have a standard deviation in running text, though is given in figures.

Done, throughout

Page 11: some comments can be added on the filtration efficiency of various masks without leakage.

Thank you for this interesting question. We have added a section on leakage to **Results>Subjective assessment** as follows:

Subjective assessment of mask leaks and glasses fogging were both significantly related to fitted filtration efficiency (p <0.05, Fig 4). Leak assessment was the stronger predictor, explaining 22% of fitted filtration efficiency, whereas glasses fogging explained 4% of variation. Trials across masks with scores of 1 (no leaks detected) achieved mean±SD filtration efficiencies of 86±12%, scores of 2 (imperfect seal to face) 74±16%, 3 (minor leaks) 71±15%, 4 (minor leaks in multiple areas) 64±15% and scores between 5-7 (major to severe leaks) averaged 60±9%. However, these observations were dependent on mask type. For example, among cloth masks tested, the range of FFEs varied by 43% (FFE range of 38-80%) across all trials whereas masks tested and given a score of 1 had mean FFEs that were improved by an average of 7% over the mean FFE of masks with leak scores greater than 5. A similar observation was noted for KF94s and KF95s, with individual masks and trials varying by 35% in FFE performance while leak scores explained about 9% of this performance difference. This indicates difficulties in perceiving leaks for these mask types. Alternatively, for Level 1 and Level 3 masks with and without hacks, perceived leak scores were much stronger predictors of mask performance. Level 1 masks exhibited a range of FFE values that varied by 51% (FFE range from 44 to 96%) across individual trials, whereas trials with Level 1 masks having an assigned leak score of 1 had FFEs between 22 and 26% higher compared to masks of this type given a leak of score of 5 or above. Similarly, leak scores assigned to level 3 masks explained as much as half of the total variation in FFEs scores observed for this mask type. All respirators were assigned the best perceived leak scores of 1 and this mask type showed the least variation in performance among mask types.

And in the **Discussion** we added:

Indeed, participants’ blinded a priori numerical assessment of mask leakage accounted for some of the variation in mask performance but this varied between mask types. Assessments about mask leakage were most effective at predicting mask performance for level 1 and level 3 masks, which likely generalizes to all certified medical masks. International standards ensure that material in these masks filters very effectively (>95%), and reduction in fitted filtration when worn is explained by leak around the mask. The finding that participants’ assessment of leak around the mask, for medical masks, explains some of the variability in leak, should be included in public health communication strategies and in instructions about the choice and use of medical masks as PPE. These are among the most commonly used masks by the public, and are widely used in health care in the management of people with COVID-19 in Canada, the UK and the European union (40-42).

Conclusion:

In conclusion, it should be mentioned that FE is based on total measured particle count and size-dependent.

We are very grateful for this important suggestion, and **have added this text to the conclusions:**

Filtration is size and weight dependent. Current standards for protection from bioaerosols derive from those for industrial protection: N95 respirators are certified and used in both contexts. The standards for N95s are 95% filtration of dense salt particles of 0.075 µm count median diameter for the material, followed by fit testing with the N95 classifier engaged, reflecting particles <0.1 µm in diameter (69-71). These standards are generally met by constructing masks from melt-blown electrostatically-modified polypropylene or other petroleum-derived plastics. Viruses, mucus and water are less dense than salt: bioaerosols are often assumed to have density similar to water at 1 g/cm3 (72), whereas the density of salt is 2.2 g/cm3 (73). (Salt particles are generated as saline but dehydrate to solid salt within milliseconds (71, 74).) Though the rigorous standards adopted from industry translate into safety for health care workers, when applied, it is not clear that these are the optimal health care standards, and new standards, equally safe, could be developed based on the filtration properties of clinically-important bioaerosols. Such re-engineering would also facilitate improvements in breathability and perhaps in sustainability, and further research in this are would also be applicable to masks worn to reduce the transmission of respiratory diseases in the community (ie, barrier face coverings).

Cloth masks and medical masks, widely used in the pandemic, filter aerosols, accounting for their partial effectiveness in reducing transmission in an airborne pandemic (9, 13, 75-78); this should inform public health messaging and advice. Medical masks are no substitute for respirators in the personal protection of health care workers, in keeping with meta-analysis of trials comparing continuous mask use with continuous respirator use (17).

________________________________________

6. PLOS authors have the option to publish the peer review history of their article (what does this mean?). If published, this will include your full peer review and any attached files.

Yes, please publish this peer review. We are very grateful to Drs Naqvi and Kumar for their expertise and helpful and supportive suggestions, which have greatly improved the manuscript.

If you choose “no”, your identity will remain anonymous but your review may still be made public.

Do you want your identity to be public for this peer review? For information about this choice, including consent withdrawal, please see our Privacy Policy.

Reviewer #1: Yes: Syed Muhammad Zaigham Abbas Naqvi

Reviewer #2: Yes: Dr. Amit Kumar

________________________________________

Thank you, Drs Naqvi and Kumar!

[NOTE: If reviewer comments were submitted as an attachment file, they will be attached to this email and accessible via the submission site. Please log into your account, locate the manuscript record, and check for the action link "View Attachments". If this link does not appear, there are no attachment files.]

While revising your submission, please upload your figure files to the Preflight Analysis and Conversion Engine (PACE) digital diagnostic tool, https://pacev2.apexcovantage.com/. PACE helps ensure that figures meet PLOS requirements. To use PACE, you must first register as a user. Registration is free. Then, login and navigate to the UPLOAD tab, where you will find detailed instructions on how to use the tool. If you encounter any issues or have any questions when using PACE, please email PLOS at figures@plos.org. Please note that Supporting Information files do not need this step.

________________________________________

In compliance with data protection regulations, you may request that we remove your personal registration details at any time. (Remove my information/details). Please contact the publication office if you have any questions.

1.	Zangmeister CD, Radney JG, Vicenzi EP, Weaver JL. Filtration Efficiencies of Nanoscale Aerosol by Cloth Mask Materials Used to Slow the Spread of SARS-CoV-2. ACS Nano. 2020;14(7):9188-200.
